# Influence of Crystal Structure, Encapsulation, and Annealing on Photochromism in Nd Oxyhydride Thin Films

**DOI:** 10.1021/acs.jpcc.1c10521

**Published:** 2022-01-24

**Authors:** Diana Chaykina, Fahimeh Nafezarefi, Giorgio Colombi, Steffen Cornelius, Lars J. Bannenberg, Herman Schreuders, Bernard Dam

**Affiliations:** † Materials for Energy Conversion and Storage, Department of Chemical Engineering, Delft University of Technology, Van der Maasweg 9, NL-2629HZ Delft, The Netherlands; ‡ Fraunhofer Institute for Organic Electronics, Electron Beam and Plasma Technology (FEP), 01277 Dresden, Germany

## Abstract

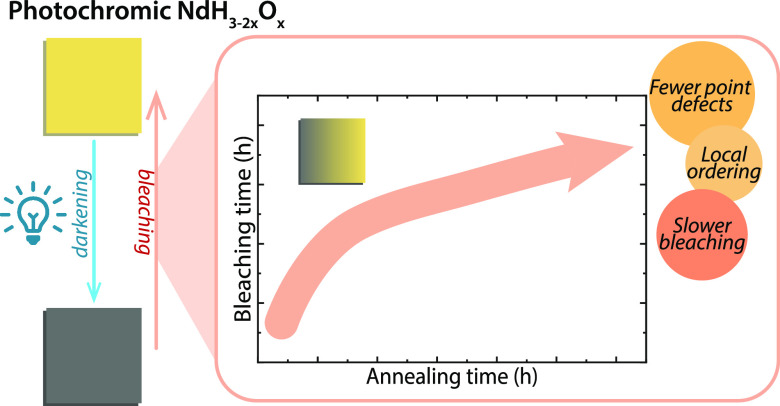

Thin films of rare earth metal oxyhydrides show a photochromic effect, the precise mechanism of which is yet unknown. Here, we made thin films of NdH_3–2*x*_O_*x*_ and show that we can change the band gap, crystal structure, and photochromic contrast by tuning the composition (O^2–^:H^–^) via the sputtering deposition pressure. To protect these films from rapid oxidation, we add a thin ALD coating of Al_2_O_3_, which increases the lifetime of the films from 1 day to several months. Encapsulation of the films also influences photochromic bleaching, changing the time dependency from first-order kinetics. As well, the partial annealing which occurs during the ALD process results in a dramatically slower bleaching speed, revealing the importance of defects for the reversibility (bleaching speed) of photochromism.

## I Introduction

Rare earth metal oxyhydrides (REH_3–2*x*_O_*x*_) thin films receive attention due to their reversible photochromic effect,^1^ where the material reversibly changes color triggered by UV light. In the presence of this incident light, the films “darken”, absorbing light over a wide range of wavelengths (visible to near-IR). Yet, when the light is removed, the original transparency is returned by “bleaching”. Such optical properties are attractive for smart window applications, especially since the bleaching speed (time required to recover the transparent state) has recently been reported as low as 9 min.^2^

Thin films of REH_3–2*x*_O_*x*_ (Sc, Y, Dy, Er, Gd) are prepared by reactive magnetron sputtering of a metallic REH_2_ film, which oxidizes to a semiconducting photochromic oxyhydride when exposed to air.^2−4^ The extent of oxidation (O^2–^:H^–^ ratio) is related to the deposition pressure during sputtering, where more oxidized films are achieved by sputtering the parent REH_2_ at a higher pressure which invokes a higher porosity of the as-deposited REH_2_. In this way, both the type of cation (RE) and the O^2–^:H^–^ ratio of these materials can be tuned, impacting their photochromic properties.^2^

Although the mechanism of photochromism in these materials is not well-defined, it has been proposed that ion mobility plays a role in the process.^2,5^ This is partly because some REH_3–2*x*_O_*x*_ powders (RE = La, Nd) have shown pure H^–^ conductivity.^6,7^ In general, these large RE cations lead to tetragonal lattices, which has sometimes been associated with anion order,^7,8^ although this last aspect is debated.^9^ Smaller RE cations, instead, result in anion-disordered cubic lattices, thus behaving as ion insulators.^7^

Because most of the reported photochromic oxyhydrides fall in the cubic ion insulator range (RE = Sc, Y, Dy, Er, Gd),^2−4^ it may be that short-range mobility, rather than long-range, influences the photochromic effect. An NMR study of YH_3–2*x*_O_*x*_, for example, showed the presence of a mobile H fraction which reversibly disappeared during photochromic darkening.^5^ However, it should be noted that other theories about the photochromic mechanism have been proposed, and not all involve a diffusion-related step, namely the formation of hydroxides, color centers, and dihydrogen species.^10^

Here, we investigate the structural properties of NdH_3–2*x*_O_*x*_ thin films and their photochromic performance. While photochromism in Nd-based oxyhydrides was reported earlier,^11^ a complete optical and structural analysis has been lacking so far. Rare earth oxyhydrides based on Nd are of particular interest because they show a high H^–^ conductivity,^7^ have a large RE cation, and have sometimes been described as anion-ordered with a tetragonal crystal structure.^7,8,12^ These structural properties of Nd oxyhydrides differ from the cubic oxhydrides we reported earlier,^2−4^ allowing for the unique opportunity to assess which structural aspects are relevant to the photochromic effect.

We find that NdH_3–2*x*_O_*x*_ thin films can be made by air oxidation of NdH_1.9+δ_ films, where the O^2–^:H^–^ ratio of the resultant film depends on the deposition pressure (*p*_dep_). However, these films are unstable in air and require a protective coating of Al_2_O_3_ deposited by ALD. Remarkably, the *c*/*a* ratio of our tetragonal NdH_3–2*x*_O_*x*_ films depends on the *p*_dep_ (or O:H ratio). While all these films are photochromic, samples made at the same *p*_dep_ showed very different color changing kinetics during photochromism, despite being equivalent in terms of crystal structure and optical properties. The variability in bleaching time is found to be due to (1) the encapsulation of the film by the protective layer and (2) the heating occurring during ALD. The former changes the order of bleaching (no longer first-order kinetics), while the latter may lead to a partial annealing of the films which eliminates some defects, slowing the bleaching time constant. This suggests that a certain “metastability” of an as-deposited REH_3–2*x*_O_*x*_ film and the associated structural defects are necessary ingredients for photochromic bleaching.

## II Experimental Section

Thin films of NdH_1.9+δ_ (∼300 nm) were deposited by DC reactive magnetron sputtering of a neodymium target (purity 99.9%, MaTecK) at 100 W in an Ar/H_2_ gas mixture at a ratio of 7:1. The vacuum system was operated at a base pressure of <10^–6^ Pa. The films were grown at various deposition pressures (*p*_dep_ = 0.3–0.9 Pa) on 10 × 10 mm^2^ fused silica (f-SiO_2_) substrates at room temperature (∼21 °C). After deposition, the films were oxidized in ambient air to form the oxyhydride (NdH_3–2*x*_O_*x*_). For comparison, some GdH_3–2*x*_O_*x*_ thin films were made by the same methods and conditions (*p*_dep_ = 0.7 Pa).

**Figure 1:**
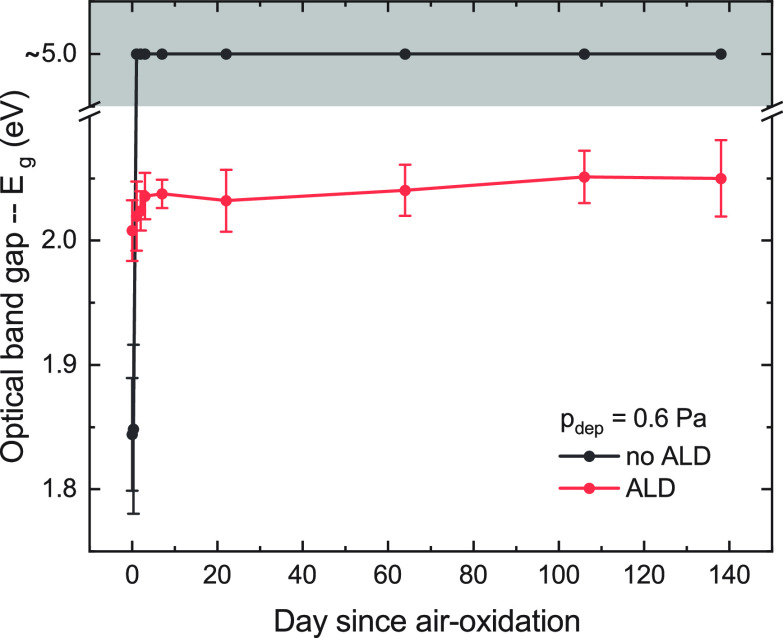
Optical band gaps for uncoated (gray) and ALD coated (red) Nd oxyhydride thin films deposited at 0.6 Pa. Day 0 is the day of deposition and removal from the vacuum chamber. The gray filled-in area indicates that the compound is fully oxidized and no longer an oxyhydride.

The Nd oxyhydride films are not stable in ambient air over long periods of time. Within a few days of removal from the vacuum chamber, the films fully oxidize (complete removal of H^–^), which is seen as a widening of the optical band gap in the transmission spectra (Figure 1 and Figure S1). To protect the films from this complete oxidation, they were coated with a conformal Al_2_O_3_ layer by atomic layer deposition (ALD) (Figure S4). After taking the as-sputtered films out of vacuum, they were brought to the ALD system, limiting the ambient air exposure during sample transport (detailed in Table SI of the Supporting Information).

The Al_2_O_3_ layers were deposited by ALD at 87 °C by using TMA (trimethylaluminum) as the precursor and O_2_ as the reactant. The TMA pulse time was set to 0.06 s, followed by a waiting time of 4 s, and an O_2_ plasma for 6 s at 300 W. The base pressure was (*p* ∼ 2 μbar), while the process pressure varied between 0.1 and 0.2 mbar. After 300 cycles (1.8 h), the ALD layer was ∼47 nm thick, determined by X-ray reflectometry (XRR) (Figure S6). ALD-coated NdH_3–2*x*_O_*x*_ films showed remarkably longer lifetimes, maintaining a stable composition (indicated by the reproducible optical transmission spectra) for at least 138 days or 5 months (Figure 1 and Figure S2). A band gap shift is noticeable in the ALD-coated films compared to the as-deposited uncoated films (day 0) (Figure S3), which may be due to slight oxidation from the combination of O_2_ plasma and heating during ALD, an effect that likely disappears as more monolayers are deposited.

Optical transmission spectra were acquired by a custom-built optical fiber spectrometer containing a deuterium and a quartz tungsten halogen lamp (DH2000-BAL, Ocean Optics B.V.) and a Si array wavelength-dispersive spectrometer (HR4000, Ocean Optics B.V.). The transmission spectra of Nd-based thin films were measured for several days to monitor the extent of oxidation for both ALD-coated and uncoated films. The optical band gap energies of the films were calculated via the Tauc method (Figure S3 and Figure S8).

The photochromic properties of ALD-coated NdH_3–2*x*_O_*x*_ were measured by illuminating the films with a narrow wavelength LED (λ = 385 nm) for 1 h and measuring the average transmittance (λ = 450–1000 nm) with respect to time. After 1 h the LED was turned off, and the bleaching process was measured for several hours until the original transparency was recovered. All optical measurements were taken at room temperature (∼21 °C). The photochromic effect was only measured for ALD-coated films because uncoated NdH_3–2*x*_O_*x*_ films oxidize constantly over time, preventing any reliable time-dependent measurements.

The structural properties of the thin films were analyzed by X-ray diffraction (XRD, Bruker D8 Discover) with a Cu source in grazing-incident (GI-XRD) geometry (incident angle = 3.2°, primary = 40 mm Goebel mirror with 0.6 mm slit, secondary = 8 mm motorized slit with LynxEye XE detector). Lattice constants were derived based on pseudo-Voigt fitting of each diffraction peak considering both *k*_α1_ and *k*_α2_. The evaluation of the unit cell symmetry can be misinterpreted due to the influence of thin film stress and texture on the observed XRD pattern. To investigate their presence, these properties were measured in Bragg–Brentano (θ–2θ) geometry with varying ψ angles (ψ = 0–80°) to probe crystallites of different orientation. The angle ψ describes the tilt of the sample perpendicular to the X-ray beam. Only ALD-coated NdH_3–2*x*_O_*x*_ films were analyzed by XRD since such measurements take several hours during which the uncoated samples oxidize.

## III Results

### A Optical Properties of Nd-Based Thin Films

**Figure 2:**
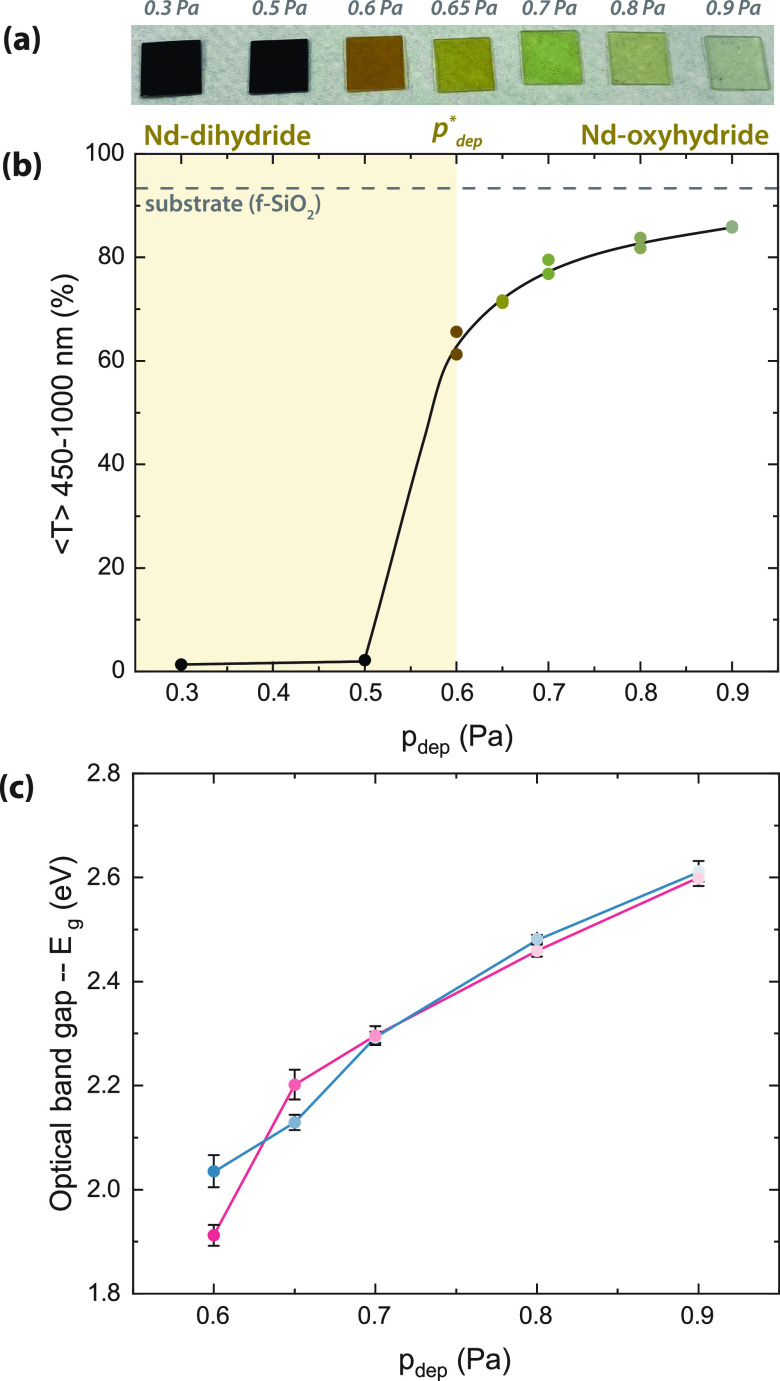
(a) Image of a set of Nd-based thin films considered for this work. They are arranged by deposition pressure (*p*_dep_) from 0.3 Pa (left) to 0.9 Pa (right). (b) Average transmittance (λ = 450–1000 nm) of NdH_3–2*x*_O_*x*_ thin films sputtered at different *p*_dep_. (c) Optical band gaps (*E*_g_) of films deposited at and above the critical deposition pressure (*p*_dep_^*^ ∼ 0.6 Pa). Pink and blue lines indicate films that showed “slow” or “fast” photochromic bleaching, respectively.

Thin films of NdH_3–2*x*_O_*x*_ deposited between *p*_dep_ = 0.3–0.9 Pa result in various optical properties upon air oxidation, similar to our previous work using other rare-earth cations (RE = Sc, Y, Gd, Er, Dy).^2−4^ Films deposited at low pressures (*p*_dep_ = 0.3–0.5 Pa) are opaque (Figure 2a), meaning low average transmittance (Figure 2b), and have no optical band gap, suggesting that they largely maintain the as-deposited NdH_1.9+δ_ composition.^13−17^

The dihydride phase of the films deposited at *p*_dep_ < 0.6 Pa is further confirmed by the small transparency window observable in the transmission spectrum (Figure S7), typical of RE dihydrides.^2−4,18,19^ The transmission spectrum for the 0.5 Pa sample, however, shows a larger transparency window, extending toward longer wavelengths. This film could be very minimally oxidized, yet still maintaining the metallic properties of as-deposited NdH_1.9+δ_.^13,14,16,17^

However, films deposited at and above a critical deposition pressure^3^ (*p*_dep_^*^ ∼ 0.6 Pa) are more transparent (Figure 2a,b) and have an optical band gap (Figure 2c). This is expected because, as the deposition pressure increases, thin films produced by sputtering are progressively more porous. Eventually, this porosity is sufficient to allow for the oxidation of the as-deposited NdH_1.9+δ_ film in air and the appearance of semiconducting properties that are characteristic of oxyhydrides (NdH_3–2*x*_O_*x*_).^2,3^

The optical band gap increases with the deposition pressure (Figure 2c) for *p*_dep_ ≥ 0.6 Pa. The relationship between the anion composition and the band gap is a phenomenon seen often in multianion compounds such as oxyhydrides,^2,4^ oxyhalides,^20^ and oxynitrides.^21^ Because the oxyhydride valence band is composed of the oxide and hydride states, and O^2–^ is more electronegative than H^–^, a replacement of H^–^ by O^2–^ shifts the valence band down.^22^ This was further investigated experimentally by using a combination of RBS and ERDA to confirm that REH_3–2*x*_O_*x*_ (RE = Sc, Y, Gd) thin films deposited at higher pressures contain more O^2–^ and have a larger band gap.^2,4^ Because the NdH_3–2*x*_O_*x*_ films described here are produced by the same methods, we expect the same trends to appear here, namely, that NdH_3–2*x*_O_*x*_ films deposited at 0.9 Pa have a larger optical band gap and O^2–^ content than those deposited at 0.6 Pa.

Notably, the band gap energies observed here for NdH_3–2*x*_O_*x*_ films (*E*_g_ = 1.91–2.61 eV) span over a wider range than what was found for other rare-earth metal oxyhydrides (REH_3–2*x*_O_*x*_, RE = Sc, Y, Gd: *E*_g_ = 2.2–2.5 eV)^2,4^ for a similar set of *p*_dep_ (Figure S8). Because the optical band gap and the O^2–^:H^–^ ratio are related, this may indicate that stable Nd-based oxyhydride thin films can be made in a larger composition range than for the other RE cations. A similar trend has been observed, for example, by Fukui et al.,^9^ where a larger stable composition range was found for La oxyhydride powders compared to Y oxyhydrides. Another possibility is that a larger spread in *E*_g_ can be generated for a similar O^2–^:H^–^ range due to the higher polarizability of Nd compared to the smaller Sc, Y, and Gd cations. Cation-based band gap engineering was shown, for example, in oxysulfides, where the conduction band was shifted by changing the RE cation gradually from Gd to Ce.^23^

### B Structural Properties

The cation size is an important determining factor for the structure of REH_3–2*x*_O_*x*_, where large cations (La–Nd) often lead to tetragonal (*P*4/*nmm*) lattices^12,24^ with anion ordering^8^ and long-range anion mobility.^7^ Smaller RE cations (Sm–Er) should then result in cubic (*Fm*3̅*m*), anion-disordered, and anion insulating materials.^7,8,25^ However, alternative structures were reported for RE = Y, La, Dy, Er, and Lu (orthorhombic *Pnma*, monoclinic *P*2_1_/*m*, and cubic *F*4̅3*m*).^9,26,27^ Eventually, though, the crystal structure of the best H^–^ conductor thus far (LaH_3–2*x*_O_*x*_)^6^ was identified as tetragonal, but anion-disordered,^9^ challenging the view that anion order is a necessity for long-range diffusion and a direct consequence of a tetragonal lattice.

Importantly, all of the aforementioned studies dealt with powder REH_3–2*x*_O_*x*_, and often only in stoichiometric compositions (REHO). For thin films, only *Fm*3̅*m* has been reported for RE = Sc, Y, Gd, Dy, and Er.^2−4,28^ The situation is less obvious for thin films of NdH_3–2*x*_O_*x*_, where some authors obtained a cubic crystal structure by epitaxy^29^ and others were not able to assign a crystal structure from XRD. Specifically, many authors use the low-intensity (101) reflection to distinguish between *Fm*3̅*m* and *P*4/*nmm* since it only appears for the latter space group.^7,8,25^ However, A∂̅alsteinsson et al.^11^ did not find this reflection for their NdH_3–2*x*_O_*x*_ films and assigned no space group. Therefore, it is unclear whether Nd oxyhydride thin films exhibit a tetragonal crystal structure as their powdered counterparts do.^7,12^

Also for our ALD-coated NdH_3–2*x*_O_*x*_ thin films, we did not observe any (101) reflection, even with careful measurement at low θ (Figure S11). This could be due to (1) the presence of a cubic lattice, (2) the inherent low intensity of this reflection (at least 10× lower intensity than (220) and (002)^7,8^), (3) an absence of anion order,^6^ and (4) thin film texture (Figure S13). To exclude the latter possibility, we performed XRD measurements tilting the film in the direction perpendicular to the X-ray beam by ψ = 0–80°. Since none of these measurements show a (101) peak, its absence is not caused by thin film texture. Because it is not immediately apparent if our NdH_3–2*x*_O_*x*_ thin films are tetragonal or cubic, we assign the reflections observed from XRD by their expected notation for a face-centered cubic lattice. Here, the (200) reflection is used to calculate “*a*” (or the (220) in the case of 0.3 Pa), while (111) is used to calculate “*c*”. In case the unit cell is truly cubic, the two values should be equal (*c*/*a* = 1). Otherwise, if *c*/*a* ≠ 1, there is a degree of tetragonal distortion.^a^

**Figure 3:**
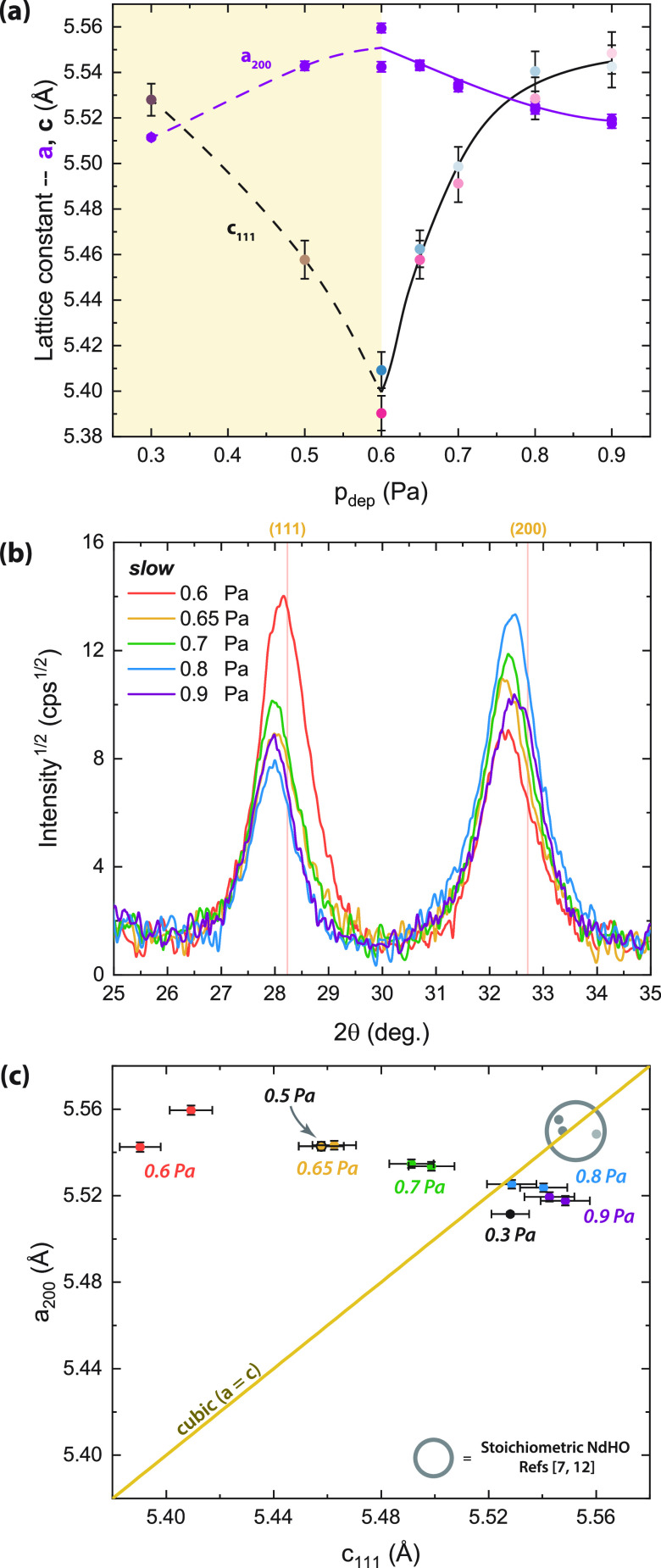
(a) Zoomed-in GI-XRD patterns of the (111) and (200) reflections for ALD-coated NdH_3–2*x*_O_*x*_ films sputtered at different pressures showing the change with O^2–^:H^–^ ratio (full patterns in Figure S10). Red reference lines are for the fcc (cubic) NdH_1.9+δ_ pattern from ICDD-PDF database # 00-89-4199. Data shown are with 2-pt smoothing. (b) Calculated lattice constants based on the (111) and (200) reflections. (c) To show the extent of tetragonal distortion, the two lattice constants are plotted against each other with a reference line for perfect cubic unit cell (*a* = *c*).

XRD patterns for films deposited below *p*_dep_^*^ are shown in Figure S9. The result for 0.3 Pa is in agreement with the fcc NdH_1.9+δ_ structure. The average lattice constant (*a* = 5.52 ± 0.01, Figure 3a) is only slightly larger than the literature value (*a* ∼ 5.46 Å).^14,15^ For 0.5 Pa, on the other hand, Figure 3a shows an expansion of *a*_200_ and a compression of *c*_111_, meaning that this film is tetragonal (*c*/*a* = 0.985). Apparently, even a minimal addition of O^2–^ is sufficient to induce a tetragonal structure.

Figure 3b shows the XRD patterns for five ALD-coated NdH_3–2*x*_O_*x*_ thin films produced at and above the critical *p*_dep_ (full patterns in Figure S10). In general, as *p*_dep_ increases, the (200) reflection shifts to larger 2θ, while the (111) peaks largely remain at the same position. Based on the calculated *a*_200_ and *c*_111_ lattice constants (Figure 3a), samples made close to the critical pressure (0.6 Pa) show a tetragonal lattice with a *c*/*a* ratio of ∼0.973, while those made at 0.9 Pa have a ratio of ∼1.005. Because the *p*_dep_ and O:H composition are related,^2,4^ we find that as more O^2–^ is incorporated into the NdH_3–2*x*_O_*x*_ lattice, the difference between *a*_200_ and *c*_111_ decreases, and the oxyhydride appears less tetragonal.

This difference in *c*/*a* and the tetragonal distortion is further highlighted in Figure 3c where the two lattice constants are plotted together with a reference line for a perfect cubic lattice (*a* = *c*). The 0.3 Pa sample is close to the cubic line, in accordance with the notion that it is NdH_1.9+δ_. Nd oxyhydride samples made at 0.8–0.9 Pa also tend toward the cubic line, while all the others are clearly tetragonal (*a* > *c*). Therefore, by changing the deposition pressure, we can produce NdH_3–2*x*_O_*x*_ films of slightly different crystal structures.

In Figure 3c, we also compare our samples to the stoichiometric NdHO powders reported in refs (7 and 12). Our values for *a*_200_ are in agreement with those of stoichiometric NdHO, but our *c*_111_ is consistently smaller. Although substrate clamping of the thin film can prevent complete expansion during air-oxidation, we found no residual stress in our films (Figure S14 and Table SII) and no significant peak shifts during heating of the films for ∼30 h at 87 °C (Figure S16), suggesting that the tetragonal distortion *c*/*a* ≠ 1 we observe is an intrinsic material property.

We further note that some of our films are more tetragonally distorted than the literature reports, with a minimum *c*/*a* of 0.973 for 0.6 Pa, while Widerøe et al.^12^ and Ubukata et al.^7^ report 1.000 and 0.998–1.000, respectively. This could be due to the composition, where films produced at *p*_dep_ > 0.8 Pa tend toward a stoichiometric NdHO composition, while all the others are H-rich. This aligns with our previous work^2,4^ where we show that our photochromic REH_3–2*x*_O_*x*_ thin films produced by air oxidation of a sputtered dihydride generally encompass the H-rich regime of the REH_3_–RE_2_O_3_ composition line.^22^ We therefore consider that the *c*/*a* ratio is a function of the O^2–^:H^–^ ratio, where less tetragonal distortion is present for a composition close to the stoichiometric NdHO, perhaps due to the decreased occupation of octahedral interstitial sites.^2,4,10,30^

At this point, it is not possible to determine if these films differ in terms of anion ordering due to the lack of neutron diffraction data. However, we assume for now that these films, similar to our previous studies,^4,22^ are anion-disordered, especially since we did not find any superstructure reflections in the XRD indicative of anion ordering. The disordered nature of the films may be due to the methods by which we produce these materials and the apparent greater stability of anion-disordered RE oxyhydrides away from the stoichiometric REHO composition.^9,22^

### C Photochromic Properties of NdH_3–2*x*_O_*x*_

**Figure 4:**
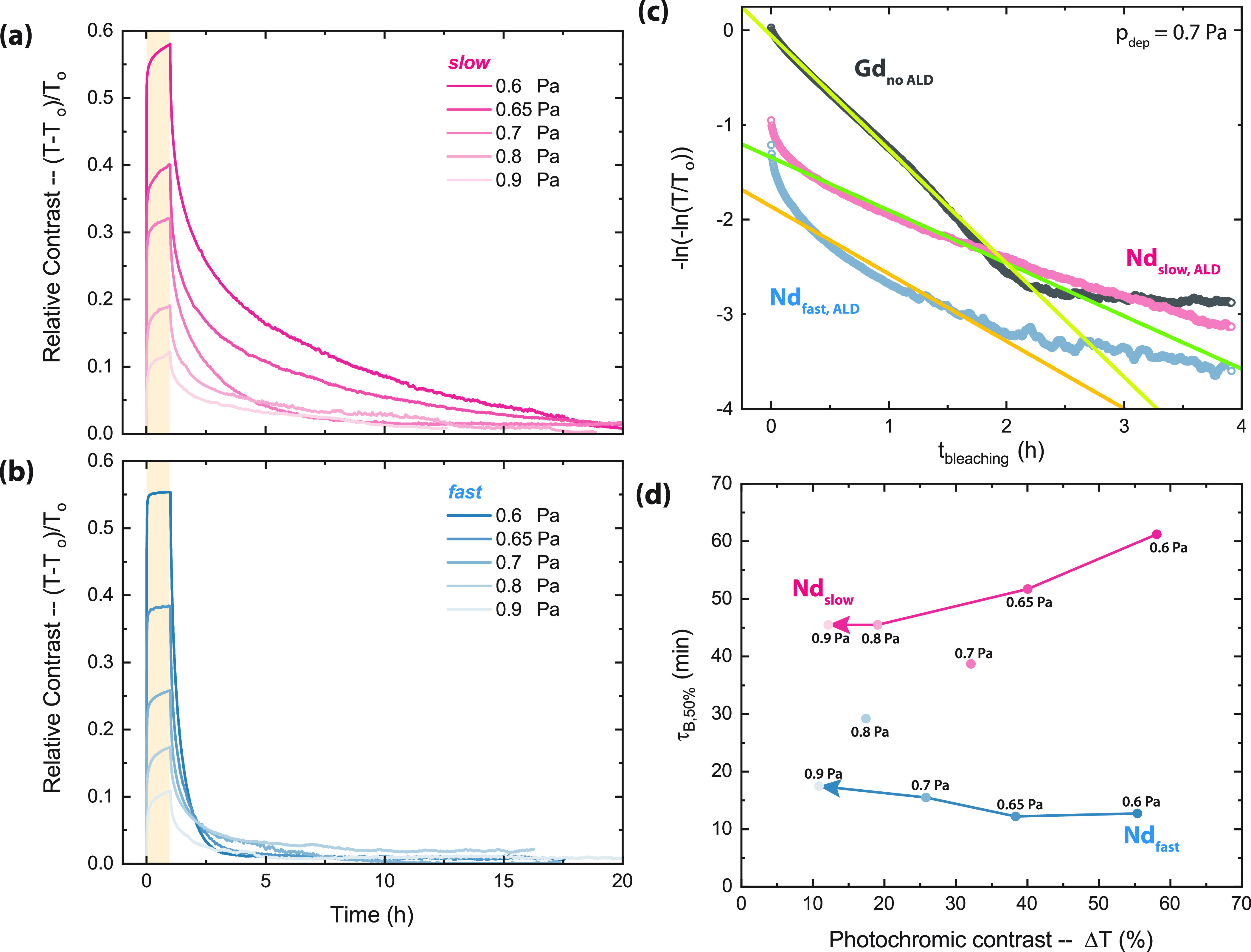
Change in relative photochromic contrast (*T* – *T*_0_/*T*_0_) over time during the photochromic effect for a set of NdH_3–2*x*_O_*x*_ thin films with (a) slow (pink) or (b) fast (blue) kinetics. The yellow box represents the time during which the samples were illuminated (1 h). (c) Double-logarithm plot normally used to derive the first-order bleaching rate constant from the linear time dependency. Only the uncoated GdH_3–2*x*_O_*x*_ film (black) shows the expected linear trend, while the coated NdH_3–2*x*_O_*x*_ films (pink, blue) cannot be described by this kinetic model. (d) Photochromic contrast (after 1 h of illumination) and bleaching time for all the samples shown in (a, b). Labels indicate the deposition pressure.

REH_3–2*x*_O_*x*_ thin films (RE = Sc, Y, Gd, Dy, Er) have photochromic properties, where the films darken during UV-light exposure and bleach back to their original transparency when the light is removed. The relative photochromic contrast ((*T* – *T*_0_)/*T*_0_) over time for our NdH_3–2*x*_O_*x*_ films is shown in Figure 4a,b. Darkening occurs for 1 h by using light with energy greater than the band gap (λ = 385 nm), which increases the relative contrast as the film becomes darker. The maximum color change after 1 h is called the photochromic contrast (Δ*T*).

**Figure 5:**
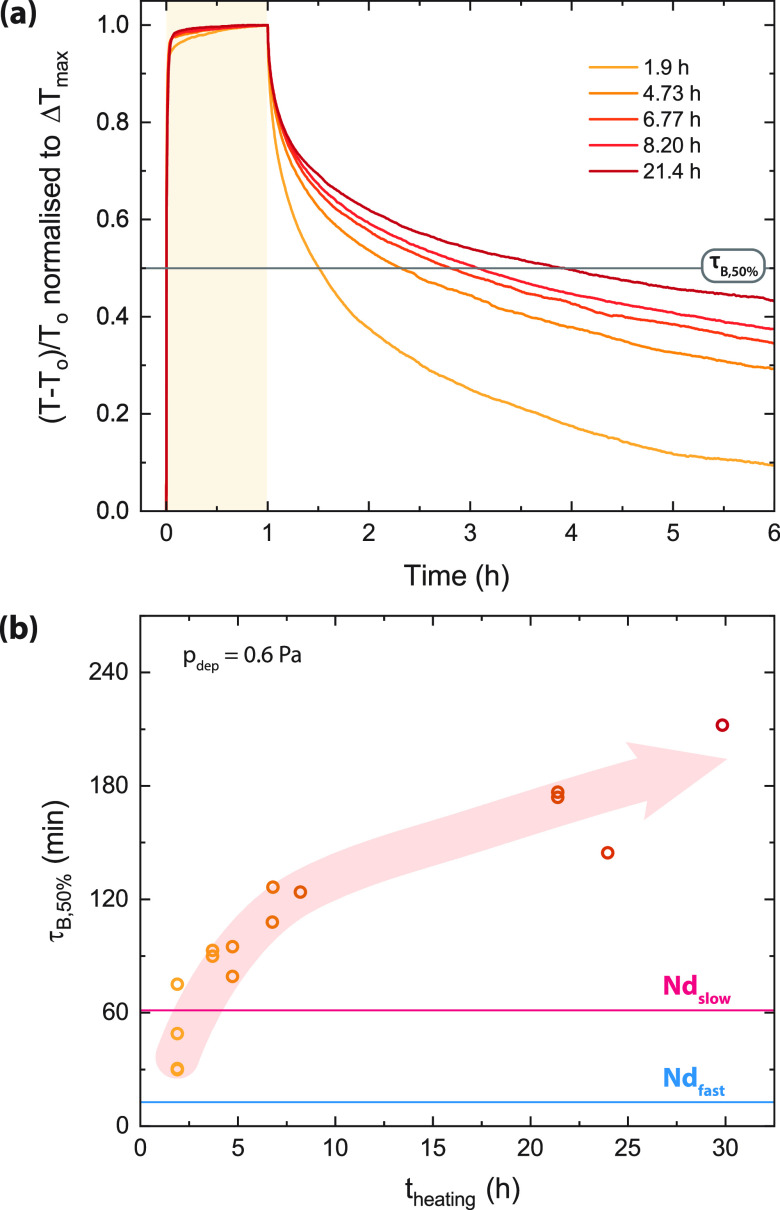
(a) Relative photochromic contrast normalized to the maximum contrast for annealed NdH_3–2*x*_O_*x*_ films made at *p*_dep_ = 0.6 Pa. Normalization was done to better visualize the bleaching speeds of films made at progressively longer *t*_heating_. (b) Bleaching time constants (τ_B,50%_) for several NdH_3–2*x*_O_*x*_ films made at *p*_dep_ = 0.6 Pa with controlled heating times. The pink and blue lines indicate the “slow” and “fast” samples discussed in Figure 4.

All of the NdH_3–2*x*_O_*x*_ films measured and presented in Figures 4a,b were coated by a protective ALD layer. The addition of this ALD coating appears to change the nature of the bleaching kinetics such that our typical expression for the rate of change during bleaching based on first-order kinetics (τ_B_)^2,3,28^ is no longer valid. This is visible for ALD-coated NdH_3–2*x*_O_*x*_ (Figure 4c) and ALD-coated GdH_3–2*x*_O_*x*_ films (Figure S15) when compared to a Gd-based film without the coating. If the assumption of first-order kinetics is correct, a linear time dependency should be visible in Figure 4c; this is only true for the *uncoated* GdH_3–2*x*_O_*x*_ film. Therefore, for this work, we define a new value, τ_B,50%_, which is the time required to lose 50% of the darkened contrast (gray line in Figure 5a).

We have shown earlier that the photochromic efficiency of a REH_3–2*x*_O_*x*_ thin film depends not only on the RE cation but on the *p*_dep_ (O^2–^:H^–^ ratio) of the film.^2^ Briefly, films made at higher *p*_dep_ resulted in a higher O content, lower Δ*T*, and faster τ_B_. Compared to the photochromic contrast of ALD-coated Nd-based films, this expected *p*_dep_-dependent trend is reproduced, since the largest contrast appears for *p*_dep_ = 0.6 Pa and the lowest for 0.9 Pa.

On the other hand, the bleaching speed (τ_B,50%_) does not follow any specific trend, and we find a wide array of values (Figure 4d). While we can distinguish between “slow” and “fast” samples (Figures 4a and 4b), the bleaching times do not show a dependence on *p*_dep_. The irreproducibility of the bleaching time can also be observed in GdH_3–2*x*_O_*x*_, used here as a reference to compare photochromism in ALD-coated and uncoated films (Figure S15). Without the ALD coating, the bleaching speed of GdH_3–2*x*_O_*x*_ films made at the same *p*_dep_ is fairly reproducible, which changes dramatically with the addition of the coating.

We can eliminate some reasons for why τ_B,50%_ varies in such a wide range. In principle, two films deposited at the same *p*_dep_ should be identical, and in many ways they are. We compared the following properties finding, for example, two 0.6 Pa samples of NdH_3–2*x*_O_*x*_ to be identical in terms of their (1) band gaps (O^2–^:H^–^ ratios) (Figure 2c), (2) crystal structure and lattice constants (Figure 3c), and (3) thin film stress and texture (Figures S13 and S14).

Instead, we studied the procedure used to deposit the ALD coating which requires heating of the films to 87 °C for a minimum of 1 h 48 min along with a few minutes of transfer time between the vacuum and air (Figure 5a). Because our samples are normally deposited, oxidized, handled, and measured entirely at room temperature, this heating can cause an annealing effect that has not been observed in previous experiments. This is especially important considering that the air oxidation used for the preparation of our films is rapid, leading to a potentially “metastable” state of the film. As well, our sputtered films tend to be polycrystalline and can contain many microstructural defects. To test the effect of annealing under vacuum (*p* ∼ 2 μbar), we made several NdH_3–2*x*_O_*x*_ films at 0.6 Pa and deposited the ALD coating onto them. Some films were removed directly after the ALD procedure was completed (*t*_heating_ = 1.9 h), while the others were left in the vacuum chamber at 87 °C for additional time.

The bleaching speed (τ_B,50%_) is strongly dependent on *t*_heating_ (Figure 5b), with longer annealing times leading to progressively slower bleaching. Because annealing can affect the structure of a material, XRD patterns were obtained for these films (Figure S16). However, we find that the lattice constants (peak positions), texture (peak intensity ratios), microstrain (FWHM), and crystal structure (*c*/*a*) do not change significantly during heating, suggesting that long-range ordering that is probed by XRD is not affected by ∼30 h of heating at 87 °C, but local/short-range order may be altered. These latter aspects are then relevant to the photochromic effect. These can include, for example, reorganization of occupied and vacant interstitial sites (i.e., changes in the compositional and structural homogeneity of the films and anion ordering), partial removal of point and/or line defects, growth of grains/removal of grain boundaries, and others.

## IV Discussion

We found that our NdH_3–2*x*_O_*x*_ films are photochromic despite having a different crystal structure compared to our previous reports on other RE cations.^2−4^ RE oxyhydrides based on Sc, Y, Gd, Dy, and Er exhibit a cubic *Fm*3̅*m* crystal structure, while the Nd oxyhydrides we present here are tetragonal to varying degrees dependent on *p*_dep_. This shows that the photochromic effect is robust and not influenced by any particular symmetry aspects.

The protective ALD coating changes the kinetics of bleaching from the first-order behavior we normally find.^3,28^ We observe this effect also when comparing coated and uncoated GdH_3–2*x*_O_*x*_ films (Figure S15). Whether or not encapsulation of REH_3–2*x*_O_*x*_ thin films influences the photochromic effect is debated^31−33^ but is outside the scope of this work. We suppose that the ALD-coated films are better described by a series of processes with no single rate constant or by kinetics of a different order. Precise conclusions require more insight about the underlying mechanism of the photochromic effect, which is still missing.

Therefore, we focus primarily on the heating in the ALD chamber and the effect of this on the photochromic properties of NdH_3–2*x*_O_*x*_ films. During this heating, local/short-range changes such as reorganization of the anion sublattice, removal of line defects, and a slight growth of grains are possible. Although these changes are difficult to quantify, they can play an important role during photochromism. Several theories have been put forth to explain this effect in REH_3–2*x*_O_*x*_ thin films^10^ without unanimous consensus. However, important phenomena can be identified and assessed within the context of this work.

We note that while the bleaching speed was dramatically influenced by heating (becoming ∼6 times slower after 30 h of heating), the photochromic contrast did not show the same trend, barely changing with heating (Figure S17). Thus, although the contrast and bleaching speed have often been considered related, this does not appear to be true for ALD-coated NdH_3–2*x*_O_*x*_ films. We suggest that darkening and bleaching do not depend on the same factors. Darkening likely depends on the presence and concentration of H^–^ and O^2–^ ions in the material since neither RE hydrides nor RE oxides are photochromic, and the photochromic contrast here only depends on the *p*_dep_ (O:H ratio) (Figure S18). Bleaching, on the other hand, is more difficult in an annealed material, perhaps due to a greater stability of the optically absorbing species in a material with fewer microstructural defects.

For example, some proposed theories describe the separation of a metallic phase during darkening and remixing back into a single phase upon bleaching. The driving force for phase desegregation/bleaching can be impacted by annealing. Our as-deposited REH_3–2*x*_O_*x*_ thin films may have an inherent anion disorder, inhomogeneity, and/or overall “metastability” which may make the dissolution of the metallic phase more favorable, a property annealed away with heating. Therefore, an annealed film would retain the darkened state for a longer period of time.

Other ideas about the mechanism of photochromism involve the trapping of charge carriers by formation of H^0^ via the excitation of an electron from H^–^. For bleaching to occur, this neutral species would have to recombine with a released electron, but this may be more difficult in an annealed material if the H^0^ can diffuse very far. Another option is that H^0^ can form a “dihydrogen” molecule,^34^ where again the energetic stability of the species in the postannealed material is important.

## V Conclusion

We prepared NdH_3–2*x*_O_*x*_ thin films by air oxidation of as-deposited NdH_1.9+δ_ thin films sputtered at different deposition pressures. As the deposition pressure increases, so does the O^2–^:H^–^ ratio and optical band gap, while the photochromic contrast decreases. The films appear to be tetragonal, with the *c*/*a* ratio approaching 1 as the deposition pressure, thus the O^2–^:H^–^ ratio, increases. Although this does not influence the photochromic effect, the tunability of the crystal structure could be important for other applications such as ion mobility.

Importantly, these films are unstable in air without a protective coating of Al_2_O_3_ deposited by ALD. Although this coating increases the stability of these films from 1 up to at least 138 days, it changes the observed bleaching kinetics. The time evolution of bleaching can no longer be described by the first-order kinetics observed for uncoated films. In addition, we find that the values for the bleaching time constant become dependent on the time spent heating in the ALD chamber (temperature = 87 °C, *p* ∼ 2 μbar).

We assume that the heating which occurs during the deposition of the protective coating results in a reduced defect concentration. As the samples were left in the ALD chamber for longer periods of time, the bleaching rate became slower, suggesting that the presence of defects in the material (e.g., grain boundaries and vacancies), and the overall imperfections of the as-deposited material are important to the reversibility of the photochromic effect. The stability of the dark species in the oxyhydride matrix may determine the bleaching speed, and annealing the oxyhydride acts to stabilize the darkened state, increasing the time needed for bleaching.

## Supporting Information Available

Optical transmission, optical microscopy, AFM images, air-oxidation condition information, XRR, full XRD patterns, residual stress analysis, results for reference measurements with GdH_3–2*x*_O_*x*_, characterization of heated NdH_3–2*x*_O_*x*_ films, and comparison of some parameters to previous reports (PDF)

## Footnotes

a When the unit cell is indeed tetragonal, the (200) from cubic notation is indexed instead as a doublet of the (220) and (002) planes. Conversion from *a*_200_ to *a*_220_ can be done by *a*_220_ = *a*_200_/sin(45°). After performing this conversion, our values for *a* and *c* remain consistent with the literature.
