# Supplementary material for: Influence of Crystal Structure, Encapsulation, and Annealing on Photochromism in Nd Oxyhydride Thin Films
Source: J Phys Chem C Nanomater Interfaces. 2022 Jan 24;126(4):2276–84. doi: 10.1021/acs.jpcc.1c10521 (PMC8819653; doi:10.1021/acs.jpcc.1c10521)
Supplement: Supplementary file 1 — jp1c10521_si_001.pdf [file jp1c10521_si_001.pdf]

# Supporting Information for:
# Influence of Crystal Structure, Encapsulation, and Annealing on Photochromism in Nd-oxyhydride Thin Films

Diana Chaykina*,* Fahimeh Nafezarefi, Giorgio Colombi,
Lars J. Bannenberg, Herman Schreuders, and Bernard Dam
*Materials for Energy Conversion and Storage,*
*Department of Chemical Engineering,*
*Delft University of Technology, Van der Maasweg 9,*
*NL-2629HZ Delft, The Netherlands*

Steffen Cornelius
*Materials for Energy Conversion and Storage,*
*Department of Chemical Engineering,*
*Delft University of Technology, Van der Maasweg 9,*
*NL-2629HZ Delft, The Netherlands and*
*Fraunhofer Institute for Organic Electronics,*
*Electron Beam and Plasma Technology (FEP), 01277 Dresden, Germany*

(Dated: January 12, 2022)

**CONTENTS**

I. Optical transmission of uncoated & ALD coated $NdH_{3-2x}O_x$ films S3

II. Microscopy & imaging of ALD coating S7

III. Air-oxidation conditions for measured films S9

IV. ALD coating thickness determination S10

V. Optical transmission of $NdH_{3-2x}O_x$ films below $p^*$ S11

VI. Optical band gap comparison to other RE-oxyhydrides S13

VII. X-ray diffraction S14

VIII. Thin film texture & strain S17

IX. Reference behaviour of uncoated & coated $GdH_{3-2x}O_x$ films S21

X. Heating of 0.6 Pa films S22

XI. Photochromism & lattice constant comparison to other RE-oxyhydrides S24

References S25

* d.chaykina@tudelft.nl

## I. Optical transmission of uncoated & ALD coated $NdH_{3-2x}O_x$ films

Oxidation in ambient air is a necessary step for the formation of our $NdH_{3-2x}O_x$ films. However, likely due to the reactivity of neodymium, these films are not stable in air. This is visible by optical transmission measurements, where the transmission of a semiconductor has a characteristic shape. At low energy (long wavelength), the transparency should be high (limited only by the substrate) since this energy is not sufficient to excite carriers across the band gap. Once this threshold (band gap) is reached, however, the transparency decreases to zero and the light is absorbed.

It has been shown in previous work that the optical band gap of RE-oxyhydrides is related to the $O^{2-}$:$H^-$ ratio, where more oxidised samples lead to larger band gaps [1]. Eventually, a fully oxidised sample (e.g., $Nd_2O_3$ or $Nd(OH)_3$) have band gaps so large that they are outside the measurement range of the equipment used here.

The evolution of the optical band gaps of uncoated Nd-oxyhydride films, thus, shows that the band gap expands rapidly, and a fully oxidised film is formed within 2 days of air-exposure. Nd-oxyhydride films coated by ALD maintain a more stable optical band gap (therefore, composition) for at least 5 months.

We can compare a freshly ALD coated film to the change in transmission for an uncoated film. Although it appears that the band gap of the material expands during the ALD process, this new composition is maintained for at least 5 months.

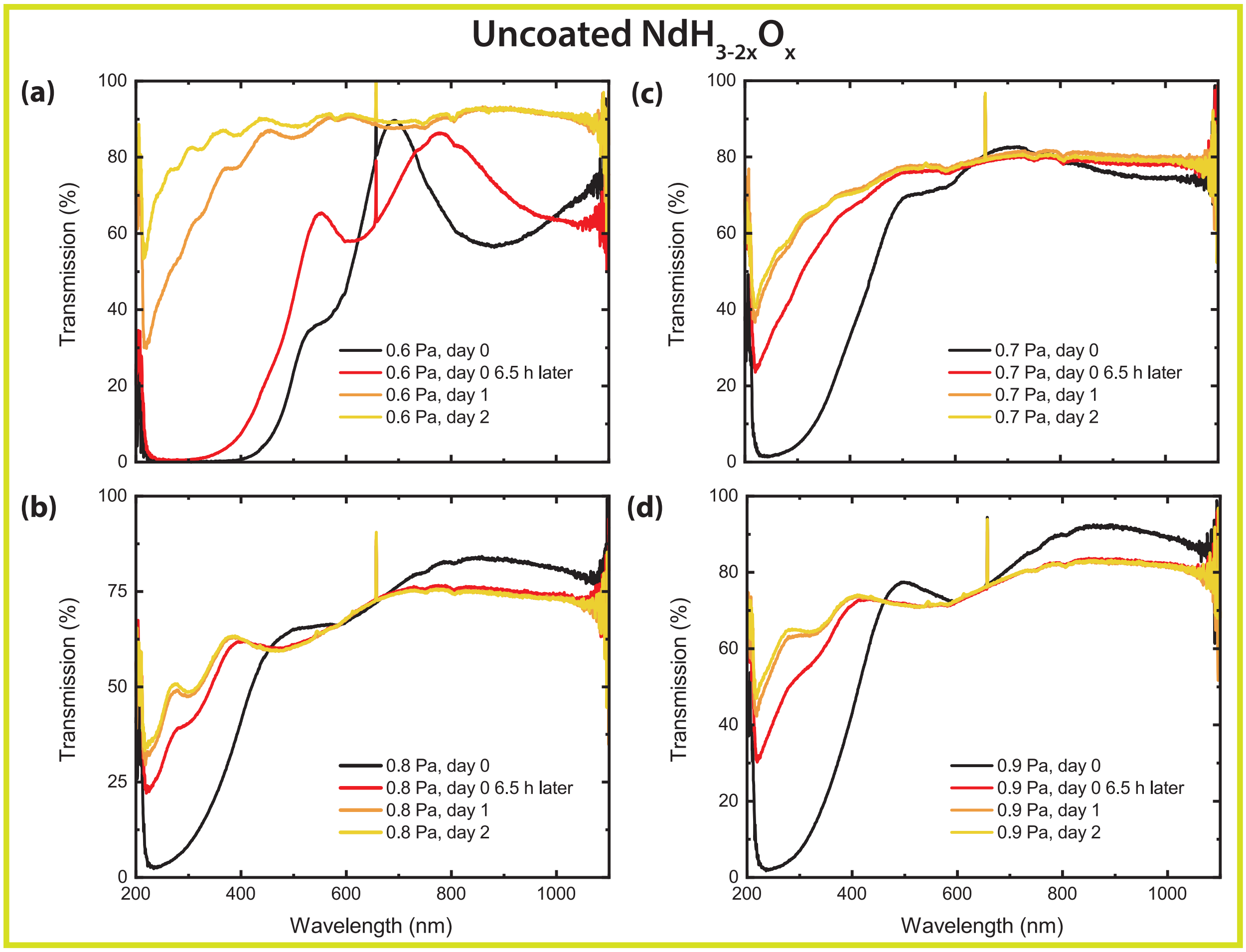


**Figure S1:** Optical transmission spectra for some Nd-oxyhydride thin films produced at different deposition pressures ($p_{dep} = 0.6 - 0.9$ Pa *without* a protective coating. Day 0 is the day of deposition and onset of air oxidation. The low transmission at short wavelengths is related to the band gap of the material. This absorption edge quickly shifts to the left (larger band gap), resulting in a completely oxidised (no $H^-$) compound by day 2 for all deposition pressures.

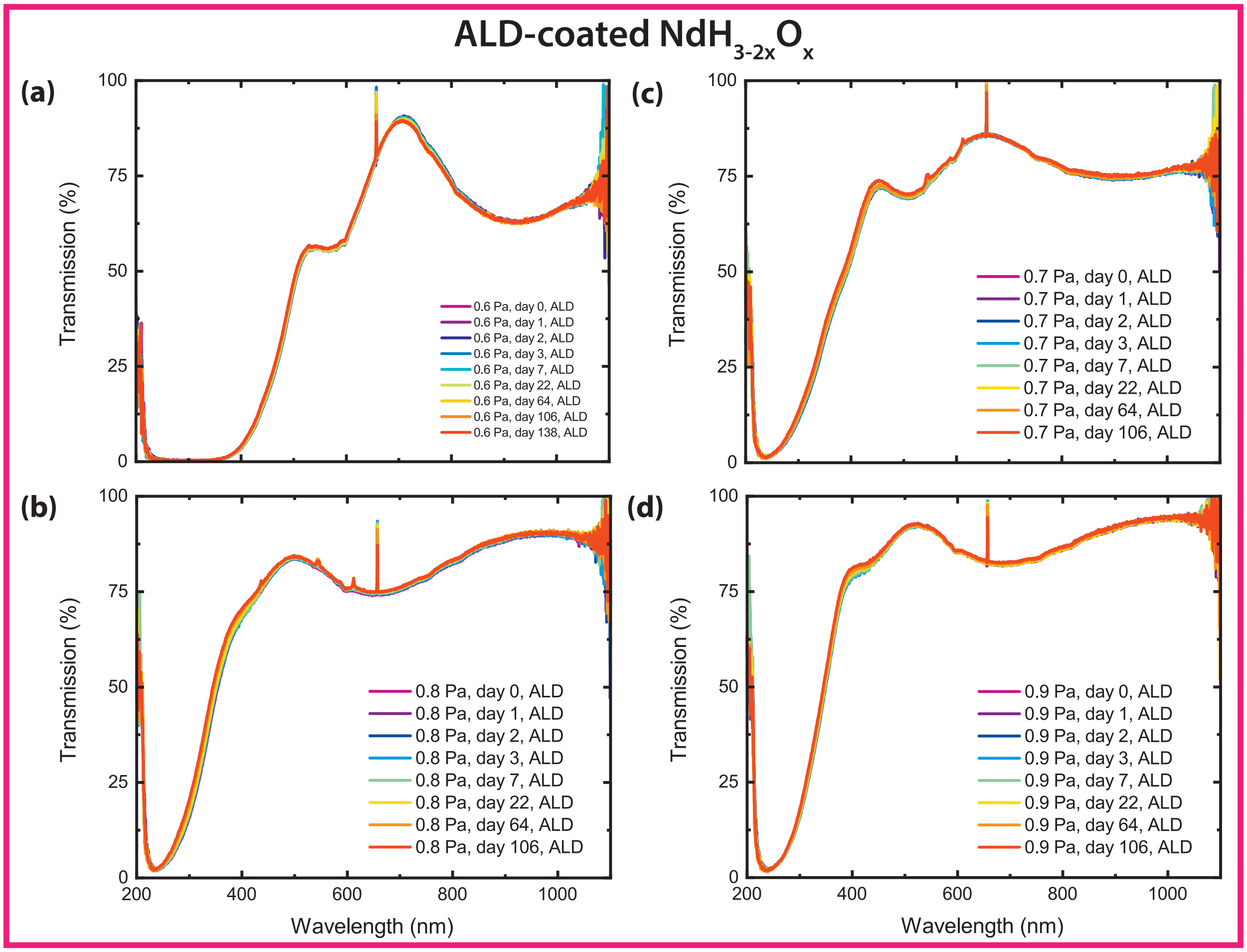


**Figure S2:** Optical transmission spectra for some Nd-oxyhydride thin films produced at different deposition pressures ($p_{dep} = 0.6 - 0.9$ Pa *with* a protective coating of $Al_2O_3$ deposited onto the films by ALD. Unlike the uncoated films, these Nd-oxyhydrides are stable in ambient air for at least 138 days since the absorption edge shows little-to-no shift over time. This implies that no signficant oxidation occurs for these films, substantially extending their lifetime.

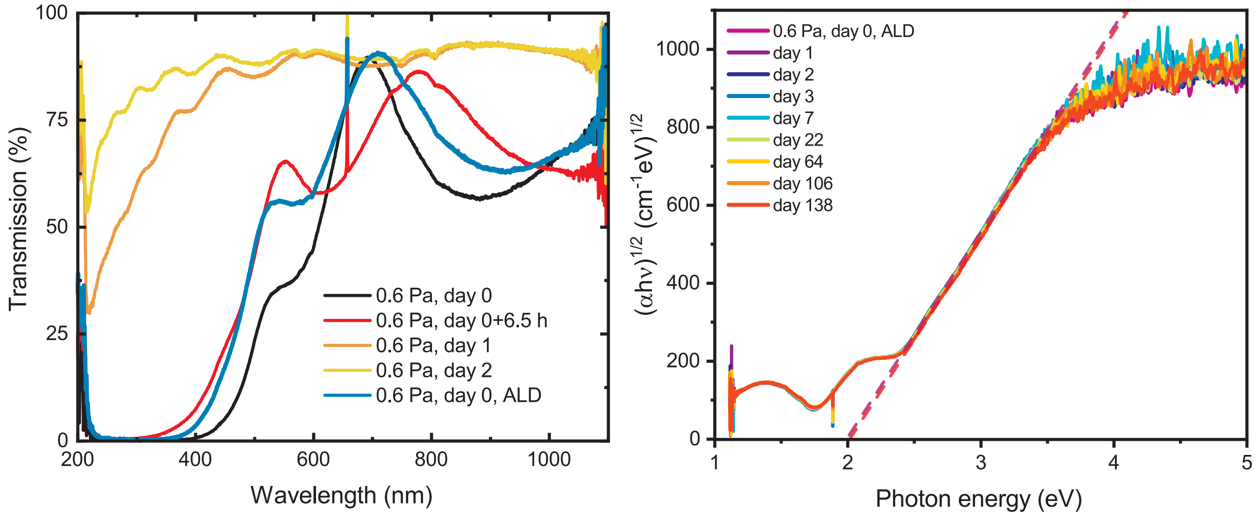


**Figure S3:** (left) A comparison of uncoated and coated Nd-oxyhydride films deposited at 0.6 Pa. The black line is the transmission spectrum of a film before ALD, and the blue line is after ALD. The band gap opens slightly during this process. Despite this oxidation, an ALD coated sample will maintain this composition for at least 138 days, unlike the uncoated film. (right) Tauc plots for the transmission data of ALD-coated Nd-oxyhydride films deposited at 0.6 Pa. Fitting lines for day 0 and day 138 are shown, where the x-intercepts (indicating the optical band gap) are similar. Shifts towards the right are related to an expansion of the optical band gap.

## II. Microscopy & imaging of ALD coating

Our $NdH_{3-2x}O_x$ films were coated by an ALD layer of $Al_2O_3$ to protect them from rapid oxidation. Below, we show the characterisation of this layer by atomic force microscopy (AFM) where we show that it is conformal (Fig. S4).

However, although this coating indeed protects our films from complete oxidation for at least 5 months, we noticed that imperfections can occur sometimes, for example, in the presence of dust (Fig. S5). The coating will deposit on the dust particle, which can fall off later and reveal either the substrate or a part of unprotected $NdH_{3-2x}O_x$. This results in pinholes in the coating which act as centres of oxidation. If there are enough pinholes, complete oxidation can take place. This is also a further testament to the positive function of the coating, without which, the samples cannot retain their composition.

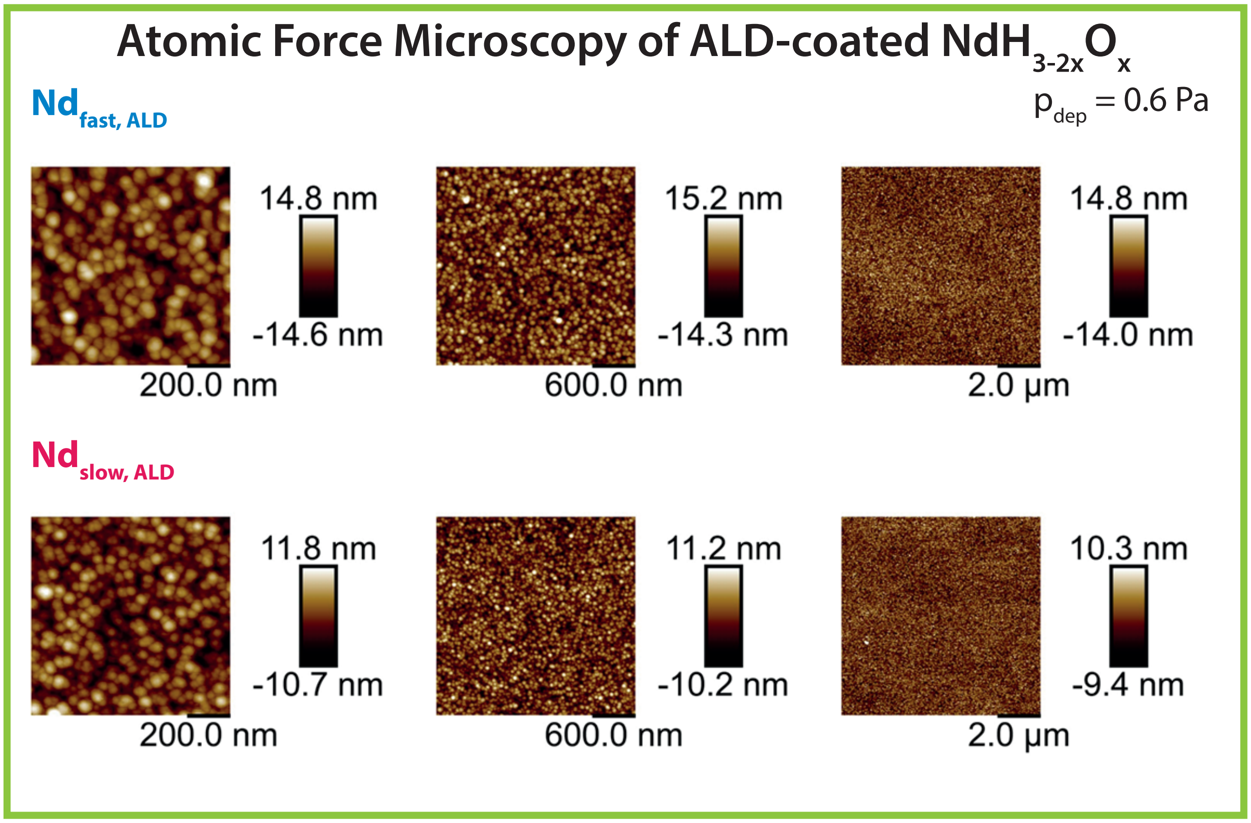


**Figure S4:** Topographic images of two $NdH_{3-2x}O_x$ films ($p_{dep}$) taken by atomic force microscopy (AFM) at different magnifications. The layer appears conformal over an area of several microns.

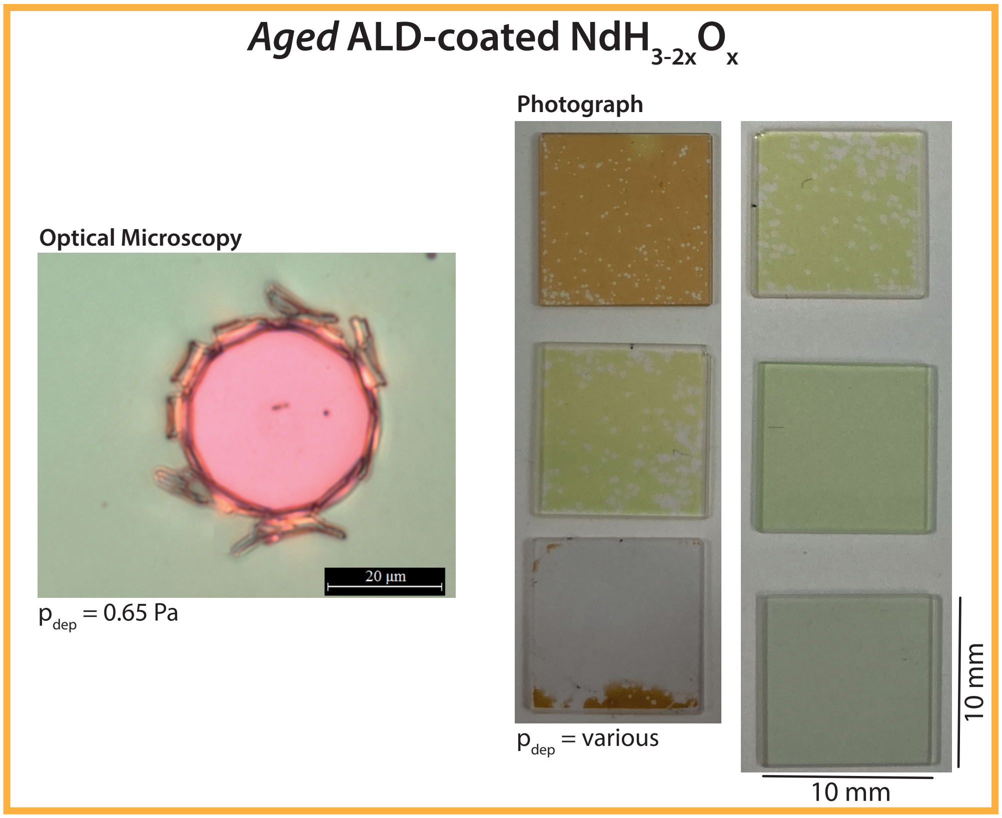


**Figure S5:** (left) Image of a pinhole in an ALD coated $NdH_{3-2x}O_x$ film ($p_{dep}$ = 0.65 Pa) taken by differential interference contrast microscopy for optimal image contrast. (right) Photographs of 10x10 $mm^2$ ALD coated samples which are several months old showing various degrees of defects in their coating and extent of oxidation.

## III. Air-oxidation conditions for measured films

**Table SI:** List of all the oxyhydride samples considered in this work. $p_{dep}$ is the deposition pressure used to sputter the sample, while "s" and "f" are used to denote which samples exhibited slow or fast bleaching kinetics, respectively. "Heat" refers to the set of samples which were treated for controlled amounts of time on the heated ALD deposition stage. The temperature is the average temperature during air-oxidation, and the transfer time is the time spent transferring the samples from the sputtering vacuum chamber to the ALD vacuum chamber (where a protective layer was deposited).

| $p_{dep}$ (Pa) | T (°C) | Transfer time (min) |
|---|---|---|
| 0.6-s | 21.4 | 2 |
| 0.6-f | 21.5 | 36.5 |
| 0.65-s | 21.6 | 3.5 |
| 0.65-f | 20.3 | 2.5 |
| 0.7-s | 20.3 | 2.5 |
| 0.7-f | 21.6 | 1.5 |
| 0.8-s | 22.1 | 2 |
| 0.8-f | 20.8 | 3 |
| 0.9-s | 20.8 | 3 |
| 0.9-f | 20.3 | 2.5 |
| 0.6-**heat** | 21.6 | 2.7 |

## IV. ALD coating thickness determination

X-ray reflectometry (XRR) was performed to determine the layer thickness of the ALD deposited $Al_2O_3$ coating. For this purpose, an $Al_2O_3$ layer was deposited under exactly the same conditions on a fused quartz substrate. The XRR measurements were performed with a Bruker D8 Discover equipped with a Cu X-ray tube (Cu-k$\alpha$, $\lambda = 0.154$ nm) and a LYNXEYE XE detector operating in 0D mode. The data, displayed in Figure S6 and fitted with GenX3 [**?** ], reveal a layer thickness of 47 nm, a roughness of 0.9 nm, and a scattering length density of 1.0 $r_e \mathring{A}^{-1}$ the $Al_2O_3$ layer, corresponding to a density of approximately 3.4 g/cm$^3$.

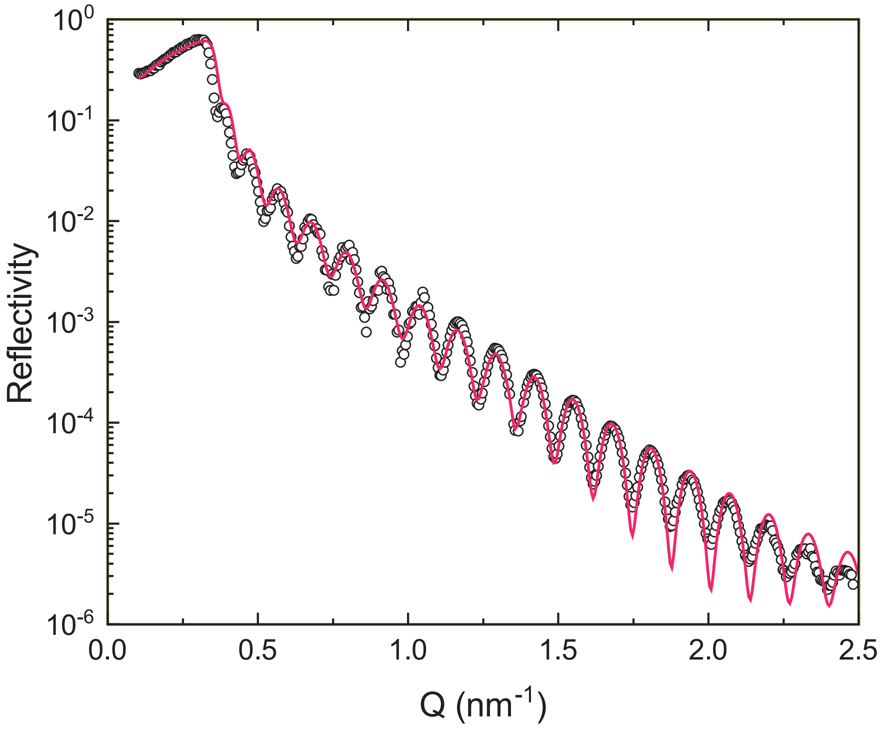


**Figure S6:** X-ray reflectometry measurement of the $Al_2O_3$ sample deposited on a fused quartz substrate under the same conditions as the $Al_2O_3$ deposited on the $NdH_{3-2x}O_x$ samples. The dots indicate the measurement points, while the continuous line indicates the fit to the data.

## V. Optical transmission of $NdH_{3-2x}O_x$ films below $p^*$

In our previous work on $REH_{3-2x}O_x$ thin films made by air-oxidation of $REH_2$, we have shown that there is a critical deposition pressure ($p^*$), above which, the oxyhydride phase is formed [1, 2]. Below we show the transmission spectra for two films made below this critical deposition pressure. Films made below $p^*$ should largely maintain their RE-dihydride composition and oxidise very minimally. This is visible especially for the ALD coated films, whose transmission spectra do not show any changes for at least 21 days. For the uncoated films, however, the transmission spectra in the initial days after removal from the vacuum chamber show very low transmission (characteristic of a RE-dihydride), but later oxidise a bit. This is true primarily for the sample deposited at 0.5 Pa which, by day 41, resembles the transmission spectrum expected for an oxyhydride.

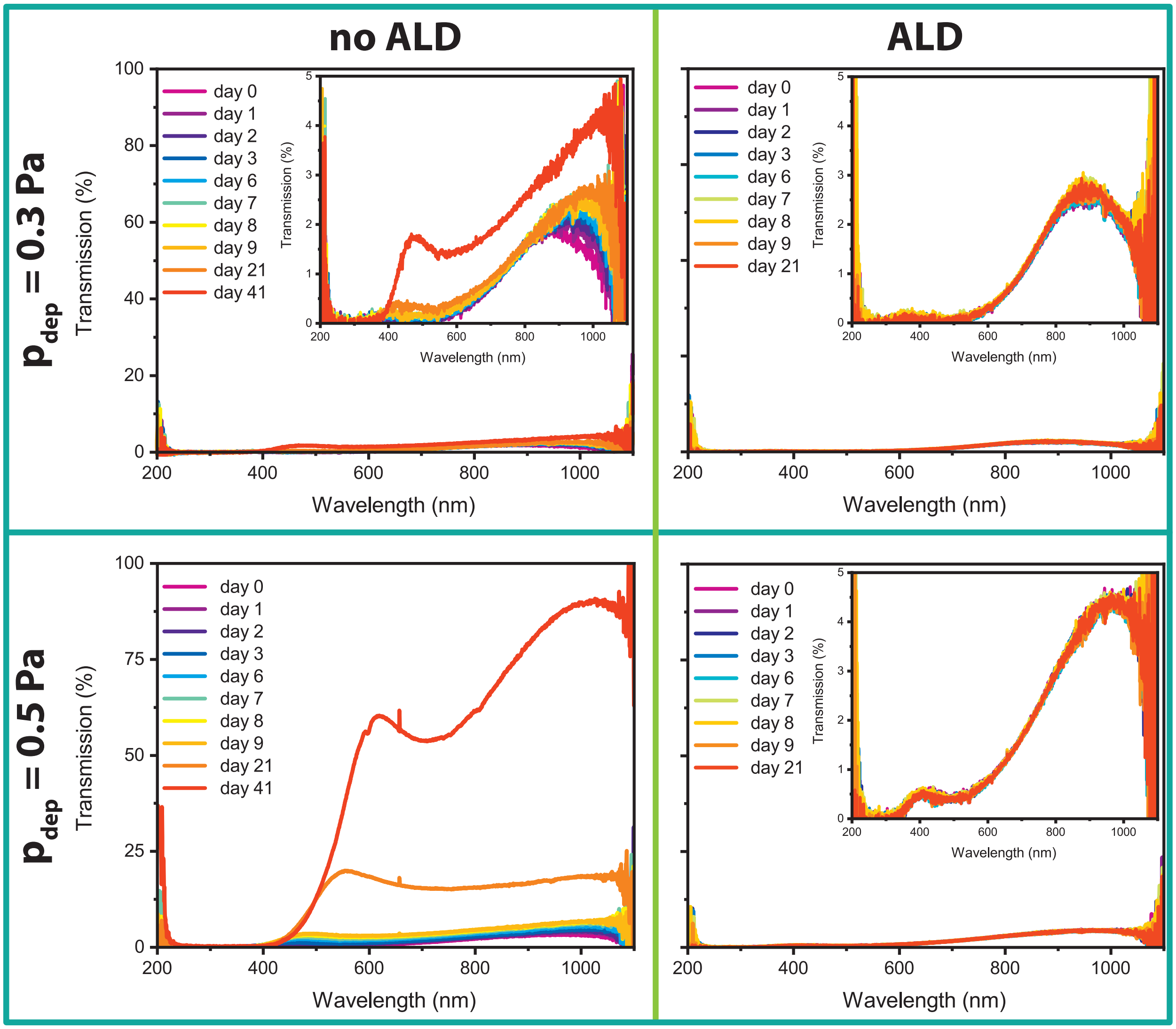


**Figure S7:** Nd-based films deposited at 0.3 (top) and 0.5 (bottom) Pa. The transmission spectra of these samples with (right) and without(left) an ALD coating were taken for several weeks after removal from the sputtering vacuum chamber. insets are provided to show the data at very low transmission. Samples coated with a protective layer retain their composition for at least 21 days, while samples without the coating progressively oxidise. This effect is more pronounced for the 0.5 Pa sample which resembles an oxyhydride by day 41.

## VI. Optical band gap comparison to other RE-oxyhydrides

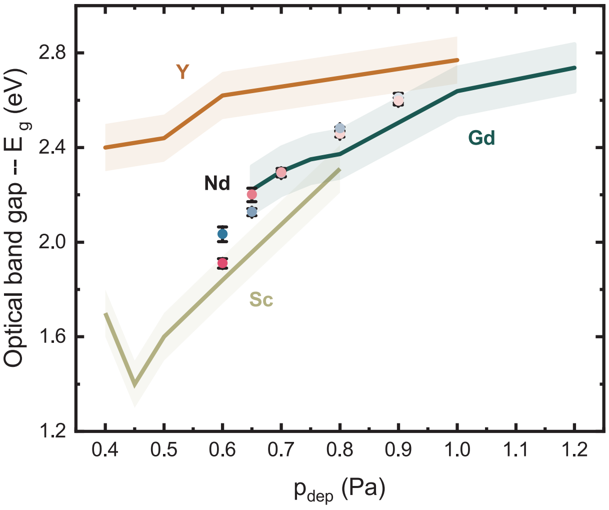


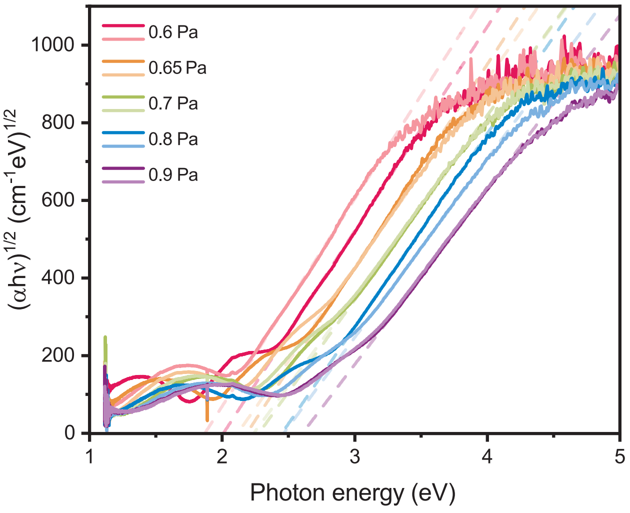


**Figure S8:** (left)The optical band gaps of Nd-based samples are shown with respect to deposition pressure, and compared to the results of Sc-, Y-, and Gd-base films from Ref. [1]. (right) Tauc plots for two samples at each sputtering deposition pressure used for this work. Fits of the linear region are shown as dotted lines and the x-intercepts are used to determine the optical band gaps.

## VII. X-ray diffraction

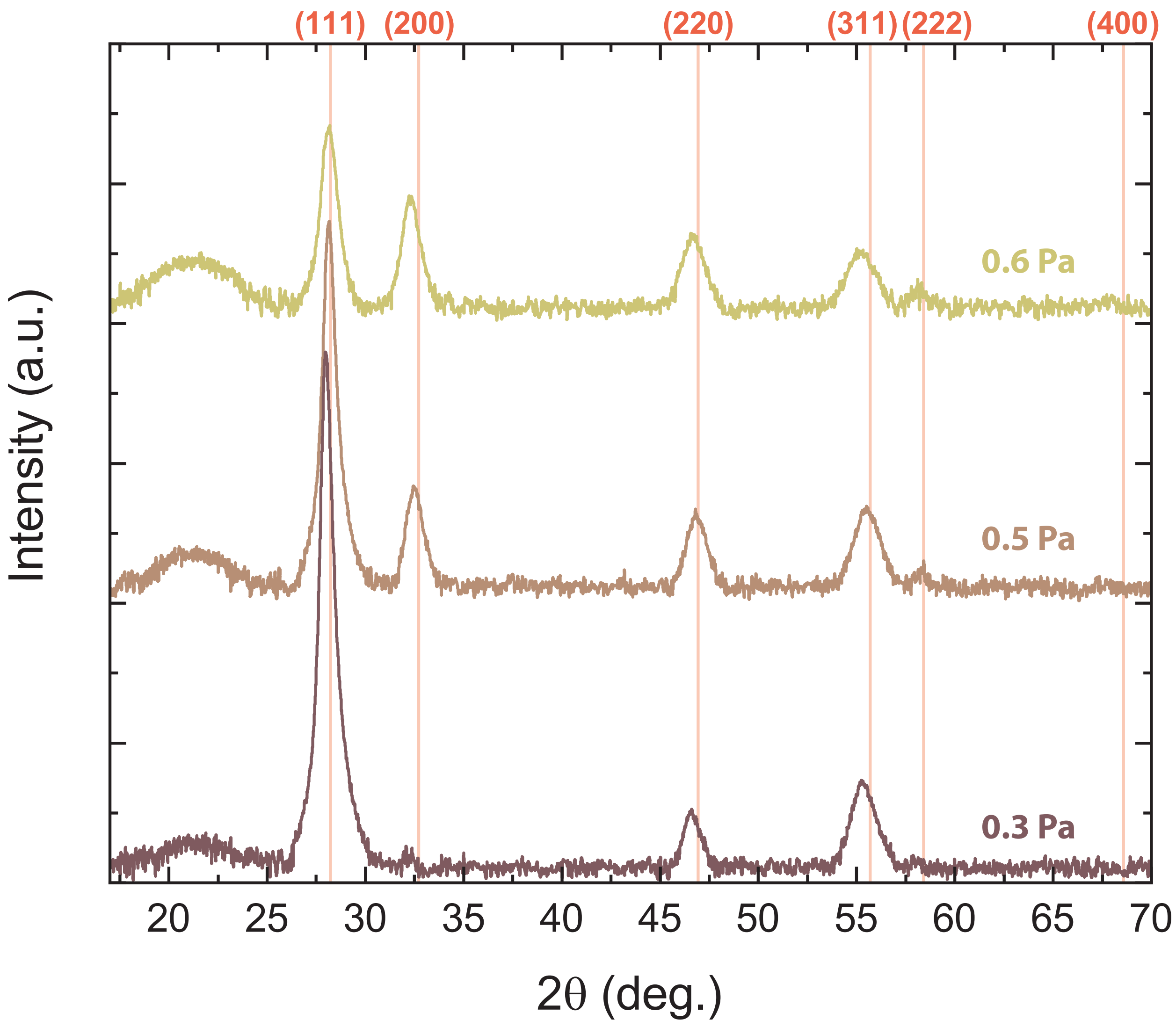


**Figure S9:** GI-XRD patterns for $NdH_{3-2x}O_x$ films made at and below the critical deposition pressure ($p^* \sim 0.6$ Pa). Samples made at 0.3 Pa resemble $NdH_{1.9+\delta}$, while an oxyhydride is obtained at 0.6 Pa. Red reference lines are for the fcc (cubic) $NdH_{1.9+\delta}$ pattern from ICDD-PDF database # 00-89-4199. The lattice of the 0.3 Pa is slightly larger than the reference pattern, and peaks are missing due to texture. The 0.5 and 0.6 Pa samples are tetragonal, thus, each peak shows a slightly different deviation from the cubic $NdH_{1.9+\delta}$ reference lines.

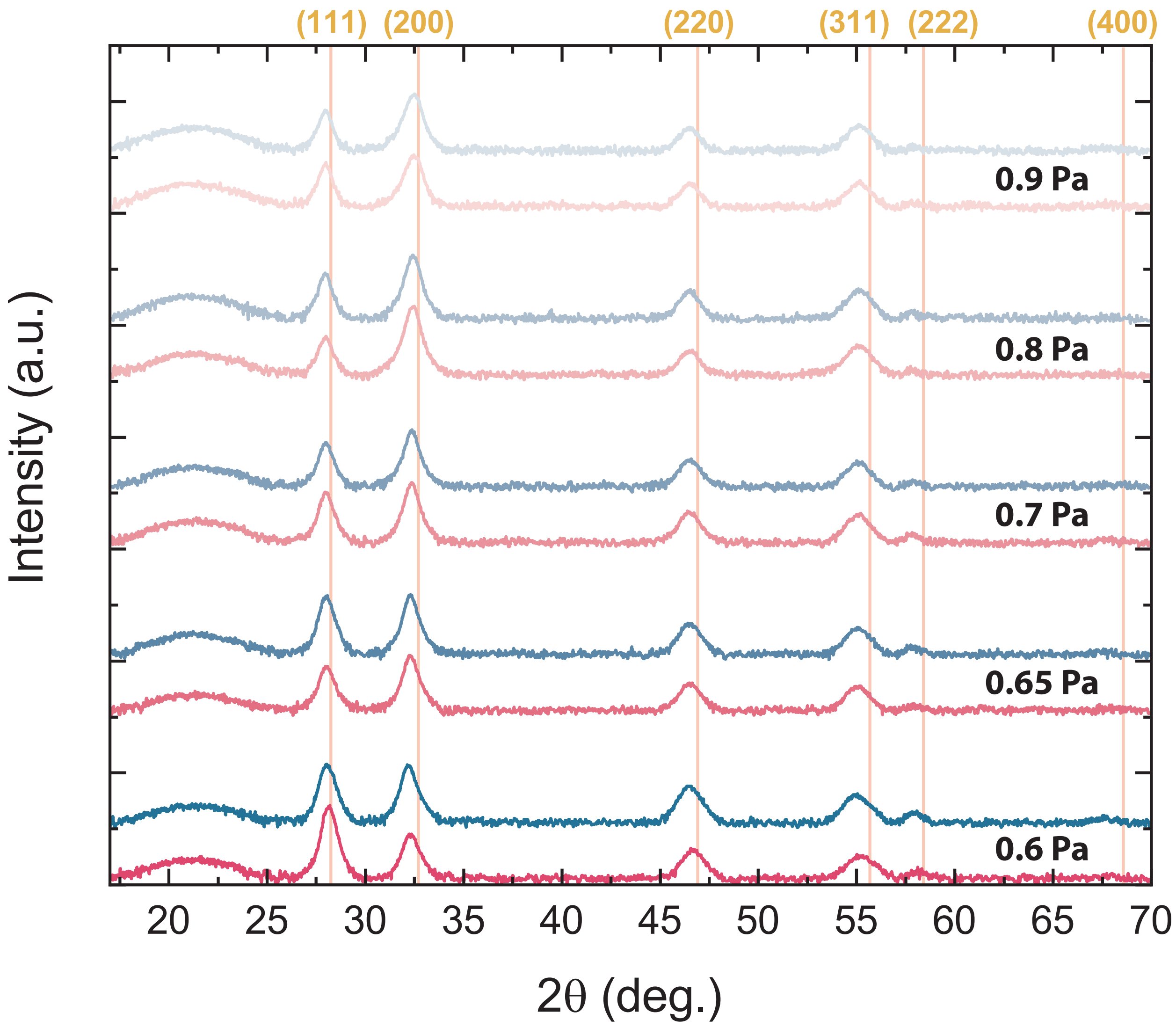


**Figure S10:** GI-XRD patterns for "slow" (pink) and "fast" (blue) bleaching $NdH_{3-2x}O_x$ films sputtered at various deposition pressures. The broad peak centred at around $2\theta = 21.5°$ originates from the underlying fused silica substrate. Red reference lines are for the fcc (cubic) $NdH_{1.9+\delta}$ pattern from ICDD-PDF database # 00-89-4199. The (111) and (200) peaks, for example, deviate from the cubic $NdH_2$ reference lines to different extents due to the tetragonality of these samples.

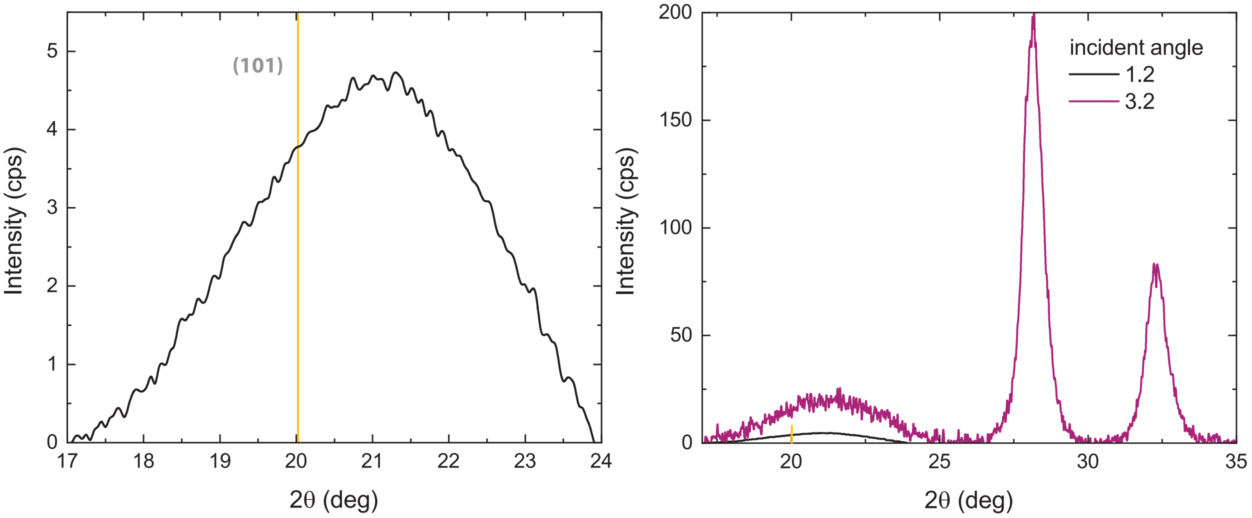


**Figure S11:** (left) GI-XRD signal obtained at low $\theta$ for a $NdH_{3-2x}O_x$ sample deposited at 0.6 Pa. The expected position of the (101) reflection is indicated by a yellow vertical line. This reflection is useful for discriminating between the most reported space groups for RE-oxyhydrides, $P4/nmm$ and $Fm\bar{3}m$, because it is only present for the former. However, even with careful scanning with a low incident angle (1.2°), only the signal of the underlying fused silica substrate is observable for one of the most tetragonal samples. (right) This low $\theta$ scan is compared to the data shown in Figure S10 for the same sample. According to the literature [3], the intensity of the (101) peak should be approximately 10 times lower than that of the (200) reflection. Based on that, we would expect the (101) reflection here to have an intensity of around 8 cps, which may be hidden under the substrate signal for incident angle 3.2°, but visible for 1.2°. However, others report an even lower intensity of the (101) [4, 5].

## VIII. Thin film texture & strain

Thin films can be analysed for their texture and macro-stress by measuring diffraction patterns at different values of $\psi$. The angle $\psi$ defines the tilt of the sample perpendicular to the X-ray beam, allowing for the measurement of crystallites of different orientations. We measured four $NdH_{3-2x}O_x$ films with Bragg-Brentano ($\theta$-$2\theta$) geometry and a $\psi$ angle varying between 0-80 °. The full XRD patterns for this are shown in Figure S12. These four films can be compared to assess the influence of not only $p_{dep}$, but also $\tau_{B,50\%}$ since two of the films showed "slow" kinetics, and two showed "fast" kinetics (specified in Table SII).

Growing thin films can develop a "texture", meaning that, although the film is polycrystalline, these crystals have a preferred out-of-plane orientation. This increases the intensity of some reflections, while decreasing the intensity of others. In extreme cases, some reflections may even disappear, as we see for our $NdH_{1.9+\delta}$ film (Fig. S9).

To examine if our ALD coated $NdH_{3-2x}O_x$ films are indeed **textured**, we compared the intensity ratios of the (200) and (111) reflections as a function of $\psi$. We define the intensity ratio, $\delta$, as:

$$\delta = \frac{I_{111}}{(I_{111} + I_{200})} \tag{1}$$

where $I_{111}$ and $I_{200}$ are the intensities of the (111) and (200) reflections, respectively. The results are shown in Figure S13. In case of random orientation, we expect a value of 0.55-0.67 for $\delta$ based on the structure factor and depending on composition. We find that $\delta$ changes slightly with $p_{dep}$ which is a consequence of the differing compositions (structure factors) of these samples. As well, $\delta$ changes with $\psi$ as different planes satisfy diffraction conditions with the change in tilt. Thus, we conclude that our films are only slightly textured.

**Macro-strain** is quantified by assessing the peak shifts for various $\psi$ angles. This is done in Figure S14, where the peak shifts are displayed as $(d - d_o)/d_o$, and $d_o$ is the value at $\psi = 0°$. This can be calculated for all the reflections which appear in the XRD pattern, however, the (220) and (222) reflection intensities were too low and reliable results could not be obtained. The slopes of the lines in Figure S14 give an indication of the macro-strain that is present in our films. Notably, the determination of macro-strain by this method relies on the assumption that the stress is uniform, isotropic, and biaxial. These assumptions are not true for weakly textured films, even so, they provide some valuable insights into the microstructural properties of our films.

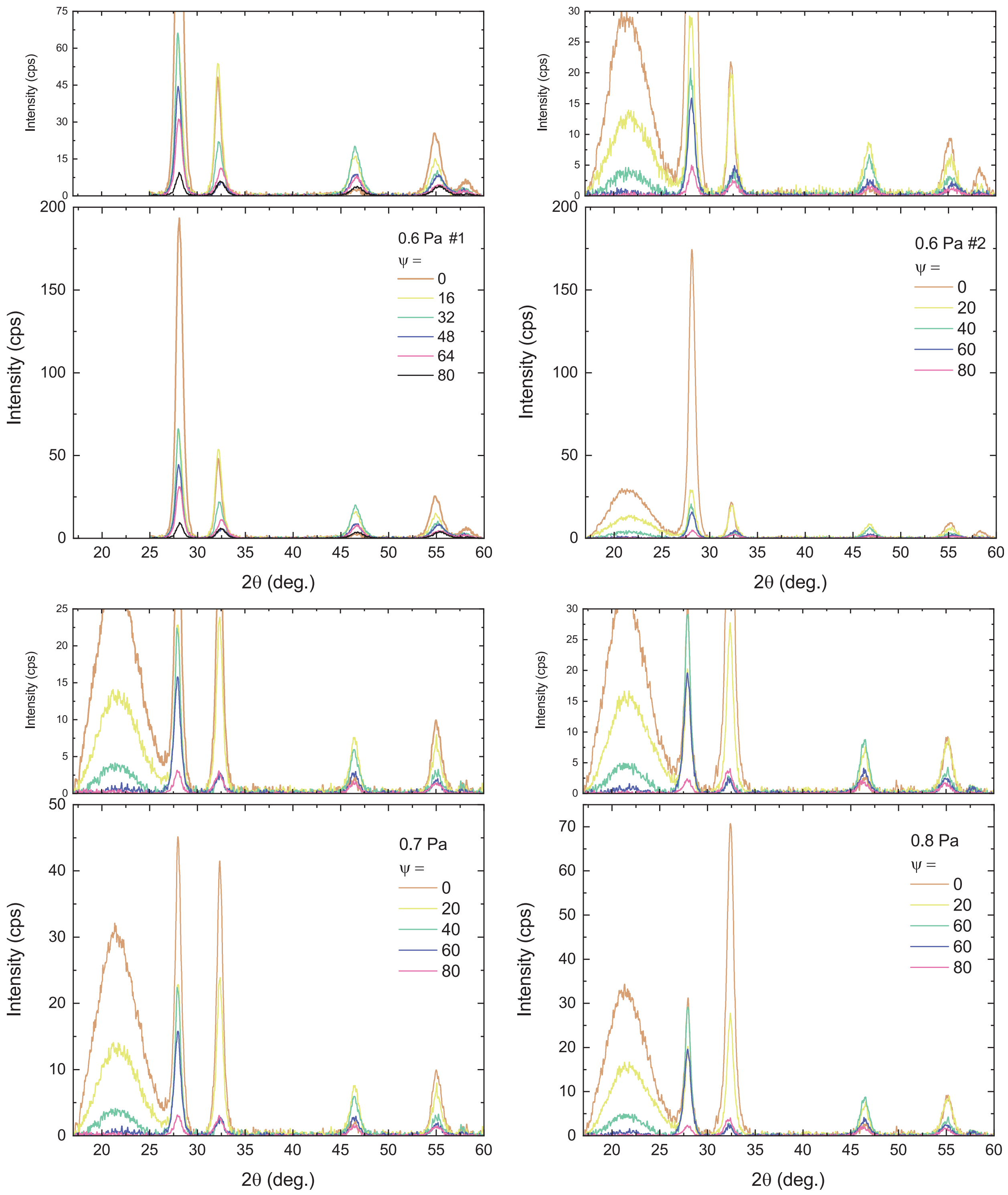


**Figure S12:** XRD patterns for the 4 $NdH_{3-2x}O_x$ films measured at different $\psi$ angles. Above each graph showing the patterns is a zoom-in version showing the low intensity peaks obtained for high $\psi$ values.

**Table SII:** Table comparing the extent of tetragonality of $NdH_{3-2x}O_x$ films to the amount of macrostrain they possess. The crystal structure of a thin film material can sometimes be misinterpreted due to the underlying stresses in the film. This can cause, for example, the assignment of a tetragonal lattice to a film which is actually cubic. However, we show here that the extent of tetragonality is always larger than the effect of macro-strain, confirming that our films are tetragonal. The macro-strain is obtained from the average of the three slopes fitted from Fig. S14, and converted to a percentage.

| $p_{dep}$ (Pa) | c/a | % tetragonality | % macro-strain |
|---|---|---|---|
| 0.6-**f** | 0.987 | 1.28 | -0.15 |
| 0.6-**s** | 0.988 | 1.23 | -0.64 |
| 0.7-**f** | 0.995 | 0.49 | 0.10 |
| 0.8-**s** | 0.996 | 0.36 | 0.30 |

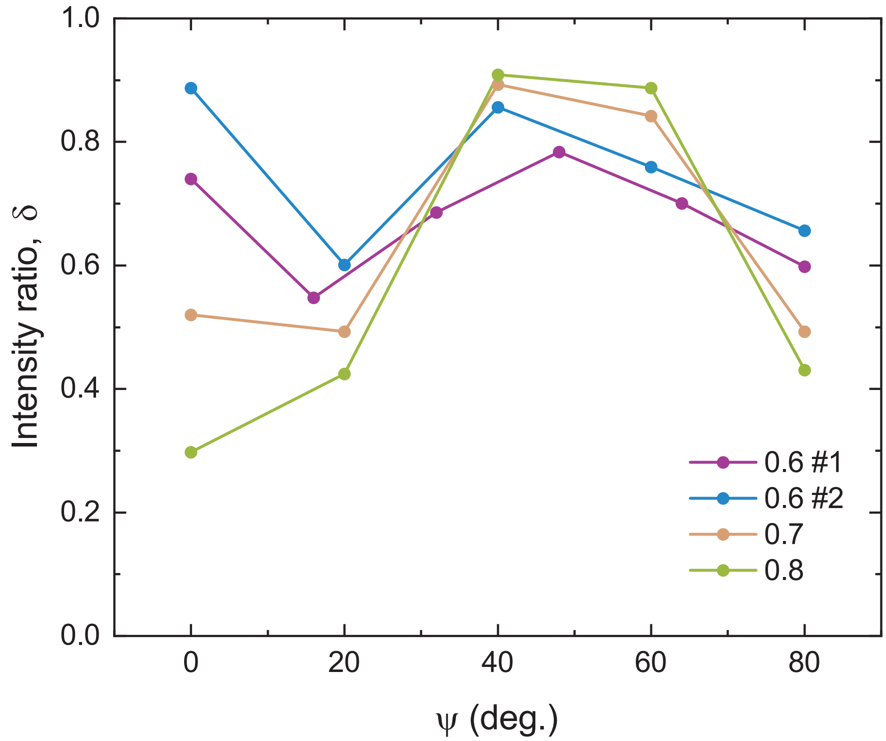


**Figure S13:** Intensity ratios ($\delta$) of four $NdH_{3-2x}O_x$ films showing a slight preferential orientation towards the (200) reflection.

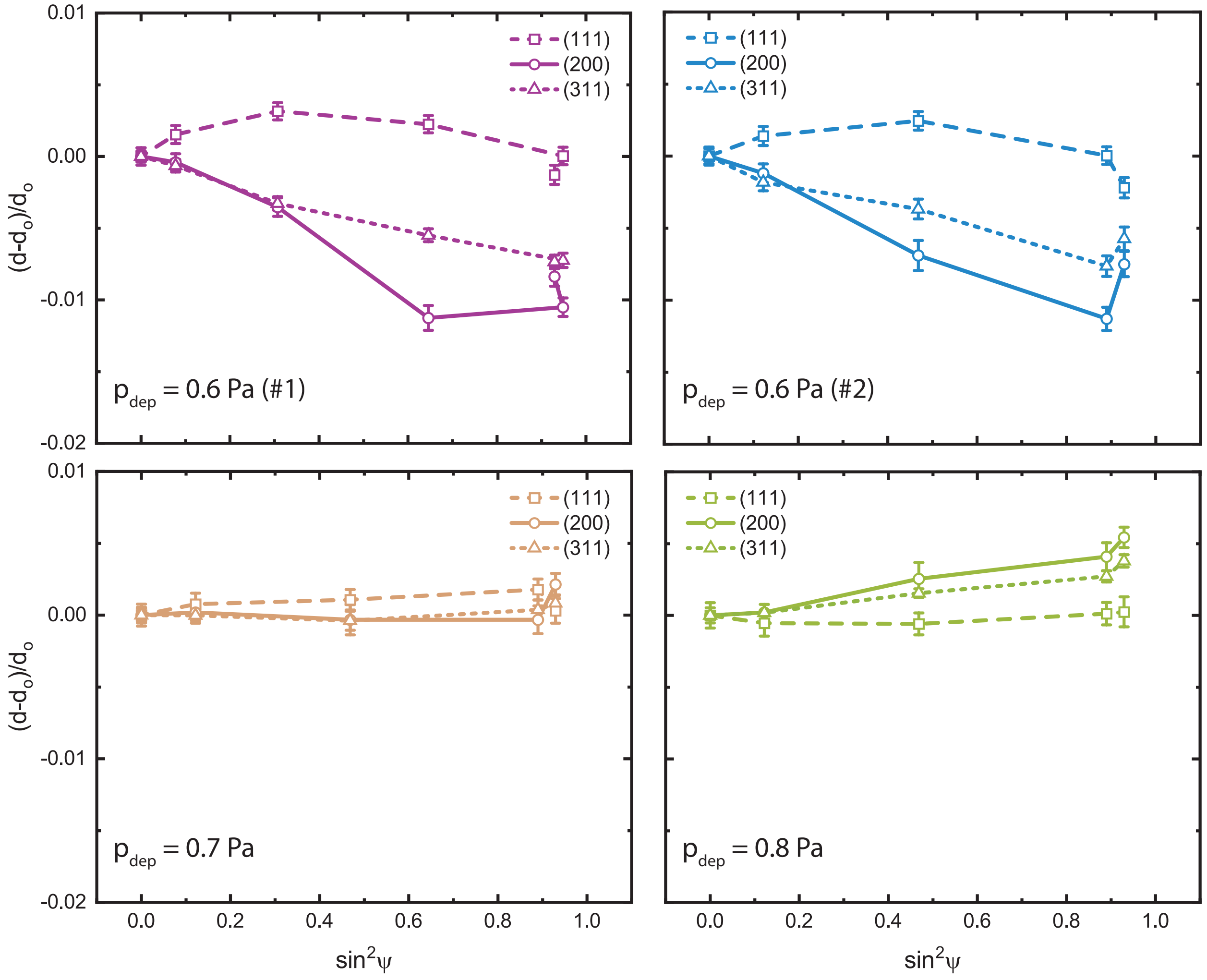


**Figure S14:** Analysis of macrostrain for 4 $NdH_{3-2x}O_x$ films. Dashed lines are derived from the (111) peak positions, solid lines from (200), and dotted lines from (311). The slopes of these lines are related to the macrostrain.

## IX. Reference behaviour of uncoated & coated $GdH_{3-2x}O_x$ films

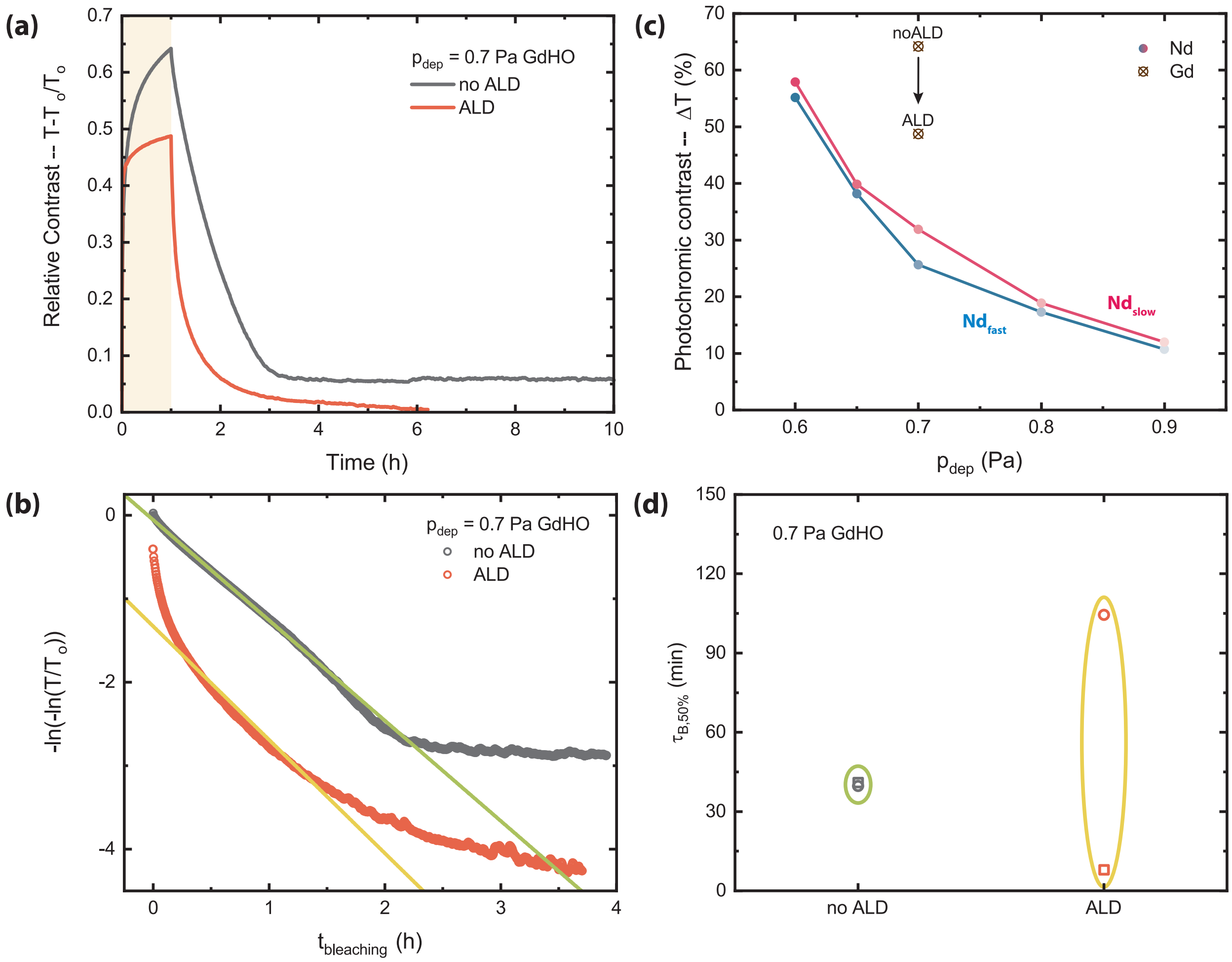


**Figure S15:** Since the photochromic effect in uncoated $NdH_{3-2x}O_x$ films cannot be reliably measured, we compare coated and uncoated $GdH_{3-2x}O_x$ films as a reference case for the effect of the protective layer: (a) Relative photochromic contrast for Gd-based films with (red) and without (grey) an ALD coating. (b) The bleaching of the coated (red) and uncoated (grey) Gd-based films shown in a double logarithm plot. Previous reports have used the slope of such a graph to obtain the negative reciprocal of the first-order bleaching rate constant [1, 2, 6]. This is in agreement with the bleaching of the uncoated sample, but does not describe the coated film. (c) Comparison of the photochromic contrast of Nd- and Gd-based films, along with the change in contrast for a Gd-based film when the ALD layer is applied. (d) The reproducibility of the bleaching time constant ($\tau_{B,50\%}$) is shown for uncoated and coated Gd-based films. The spread in values for uncoated films is very small, while for coated films, the variability is much larger.

## X. Heating of 0.6 Pa films

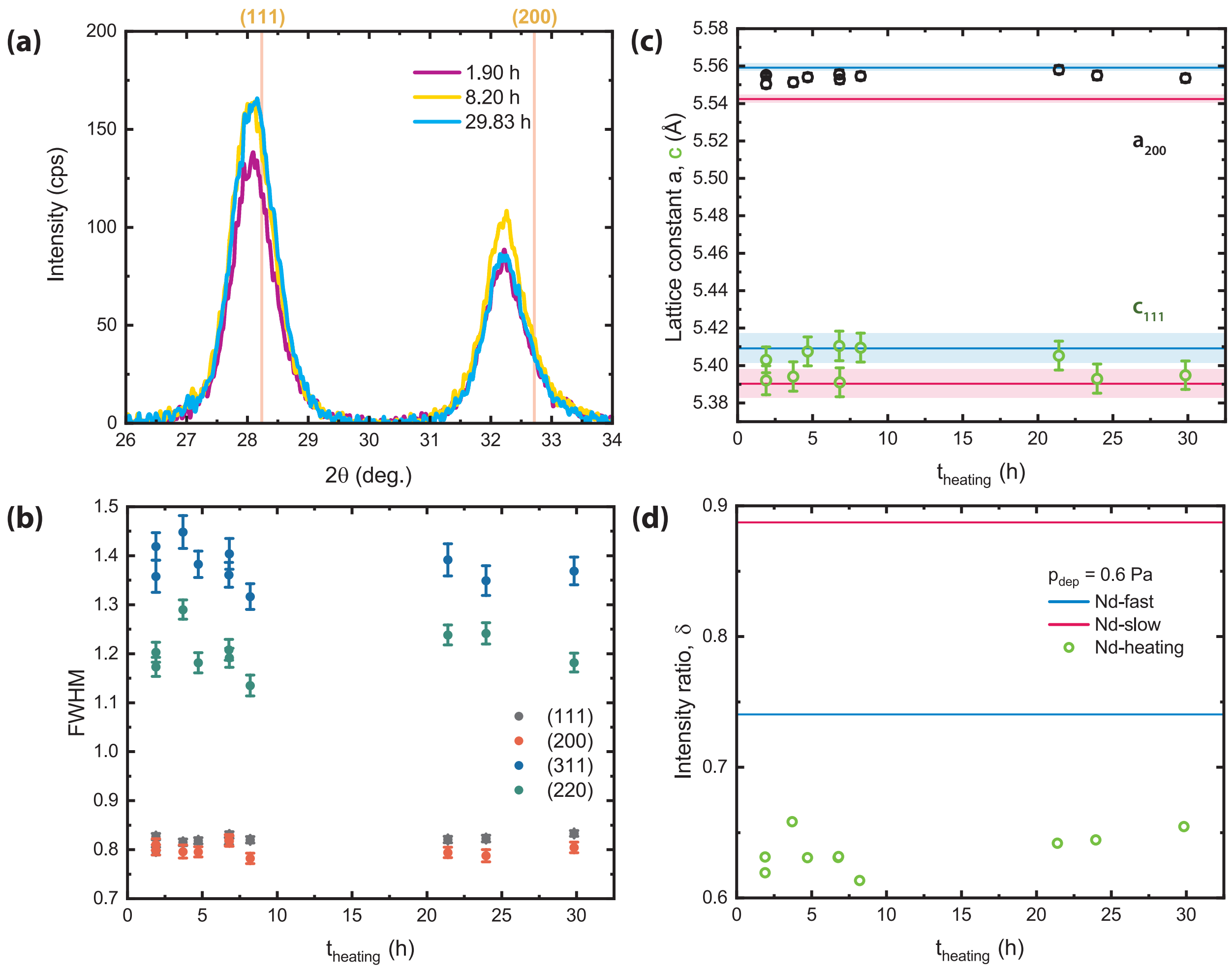


**Figure S16:** (a) GI-XRD patterns for three $NdH_{3-2x}O_x$ films ($p_{dep}$ = 0.6 Pa) heated for various times at 87°C in the ALD chamber ($p \sim 2$ $\mu$bar). Only the (111) and (200) reflections are shown. Red reference lines are for the fcc (cubic) $NdH_{1.9+\delta}$ pattern from ICDD-PDF database # 00-89-4199. Several quantities can be derived from those XRD patterns such as (b) the full-width half-maxima of all the visible reflections, (c) the lattice constants $a_{200}$ and $c_{111}$, and (d) the intensity ratio ($\delta = \frac{I_{111}}{(I_{111}+I_{200})}$). None of these quantities change significantly during ~30 h of heating at 87°C. The shaded pink and blue areas in (c) indicate the error associated with the lattice constant for slow and fast bleaching $NdH_{3-2x}O_x$ films (0.6 Pa), respectively. As well, in (d), the pink and blue lines show the intensity ratios for the slow and fast bleaching samples, respectively.

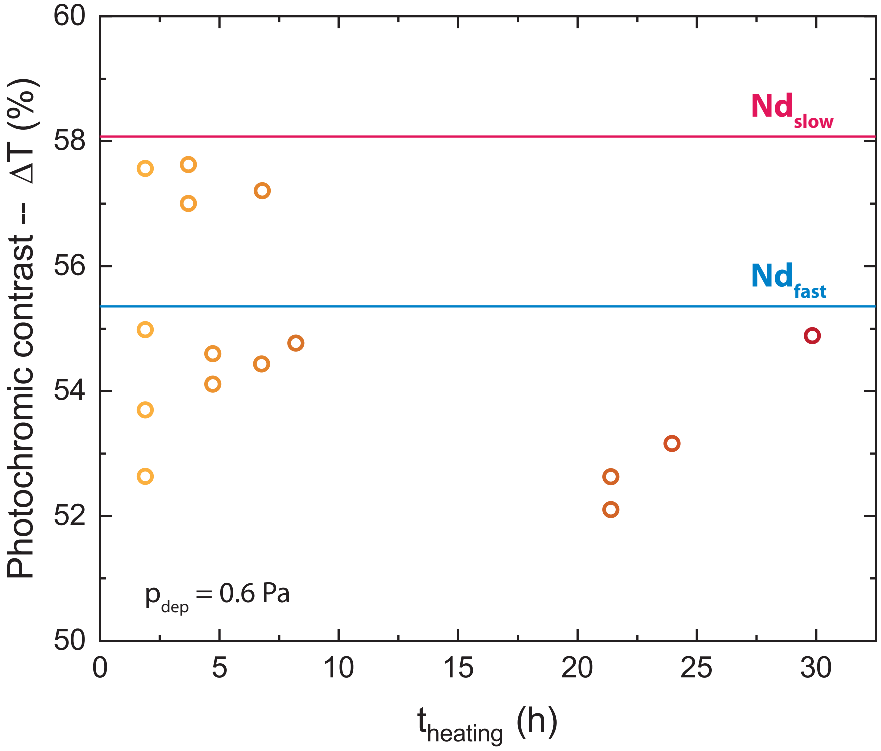


**Figure S17:** The photochromic contrast obtained for films heated for different durations in the ALD vacuum chamber. Pink and blue lines refer to the "slow" and "fast" bleaching samples presented in the main text Figure 3. No significant differences can be observed for the photochromic contrast with heating.

## XI. Photochromism & lattice constant comparison to other RE-oxyhydrides

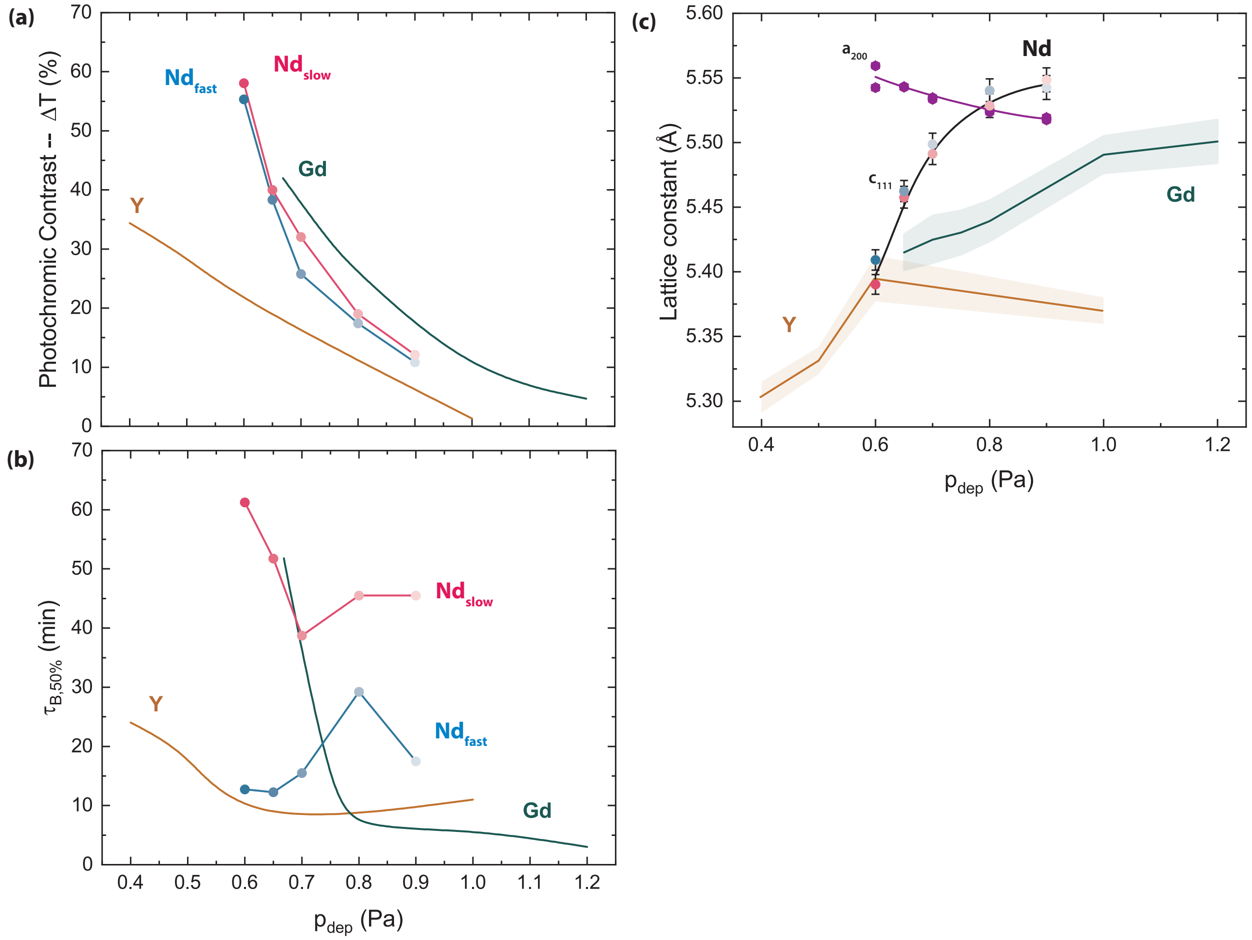


**Figure S18:** A comparison of our coated $NdH_{3-2x}O_x$ films to the uncoated Y- and Gd-based films from Ref. [1] with respect to (a) photochromic contrast, (b) bleaching speed, and (c) lattice constant. In terms of photochromism, the Y- and Gd-films were half the thickness of our $NdH_{3-2x}O_x$ films, and the illumination time was also half of what we use in this work. Additionally, the Y- and Gd-films exhibit a cubic crystal structure, so only one lattice constant (a) is given for them.

[1] G. Colombi, T. De Krom, D. Chaykina, S. Cornelius, S. W. H. Eijt, and B. Dam, Influence of cation (RE = Sc, Y, Gd) and O/H anion ratio on the photochromic properties of $REO_xH_{3-2x}$ thin films, ACS Photonics **8**, 709 (2021).

[2] F. Nafezarefi, H. Schreuders, B. Dam, and S. Cornelius, Photochromism of rare-earth metal-oxy-hydrides, Applied Physics Letters **111**, 103903 (2017).

[3] H. Ubukata, T. Broux, F. Takeiri, K. Shitara, H. Yamashita, A. Kuwabara, G. Kobayashi, and H. Kageyama, Hydride conductivity in an anion-ordered fluorite structure LnHO with an enlarged bottleneck, Chemistry of Materials **31**, 7360 (2019).

[4] M. Widerøe, H. Fjellvåg, T. Norby, F. Willy Poulsen, and R. Willestofte Berg, NdHO, a novel oxyhydride, Journal of Solid State Chemistry **184**, 1890 (2011).

[5] H. Yamashita, T. Broux, Y. Kobayashi, F. Takeiri, H. Ubukata, T. Zhu, M. A. Hayward, K. Fujii, M. Yashima, K. Shitara, *et al.*, Chemical pressure-induced anion order-disorder transition in LnHO enabled by hydride size flexibility, Journal of the American Chemical Society **140**, 11170 (2018).

[6] F. Nafezarefi, S. Cornelius, J. Nijskens, H. Schreuders, and B. Dam, Effect of the addition of zirconium on the photochromic properties of yttrium oxy-hydride, Solar Energy Materials and Solar Cells **200**, 109923 (2019).
